# Mechanochemical one-pot synthesis of heterostructured pentlandite-carbon composites for the hydrogen evolution reaction†

**DOI:** 10.1039/d3sc04542k

**Published:** 2023-09-29

**Authors:** David Tetzlaff, Tilo Rensch, Leonard Messing, Petra Banke, Sven Grätz, Daniel Siegmund, Lars Borchardt, Ulf-Peter Apfel

**Affiliations:** a Fraunhofer UMSICHT Osterfelder Straße 3 DE-46047 Oberhausen Germany ulf-peter.apfel@umsicht.fraunhofer.de; b Ruhr University Bochum, Inorganic Chemistry I Universitätsstraße 150 DE-44780 Bochum Germany ulf.apfel@rub.de lars.borchardt@rub.de

## Abstract

We have utilized carbon sources as milling additives to enable a direct mechanochemical one-pot synthesis of Fe_3_Co_3_Ni_3_S_8_/carbon (Pn/C) materials using elemental reaction mixtures. The obtained Pn/C materials are thoroughly characterized and their carbon content could be adjusted up to 50 wt%. In addition to carbon black (CB) as an additive, Pn/C materials were produced using graphite, reduced graphene oxide (rGO), and carbon nanotubes (CNTs), which allows the overall physicochemical properties of materials for energy storage applications to be adjusted. By employing the Pn/C materials as electrocatalysts for the HER in a zero-gap proton exchange membrane (PEM) electrolyzer, we were able to reach a current density of 1 A cm^−2^ at a cell potential as low as 2.12 V using Pn, which was synthesized with 25 wt% CB. Furthermore, electrolysis at an applied current density of 1 A cm^−2^ for 100 h displays a stable performance, thus providing a sustainable synthesis procedure for potential future energy storage applications. Herein, we show that catalyst supports play an important role in the overall performance.

## Abstract 2

Utilizing carbon sources as milling additives for the direct mechanochemical one-pot synthesis of Fe_3_Co_3_Ni_3_S_8_/carbon (Pn/C) materials, employing elemental reaction mixtures to adjust overall physicochemical properties.

## Graphical abstract

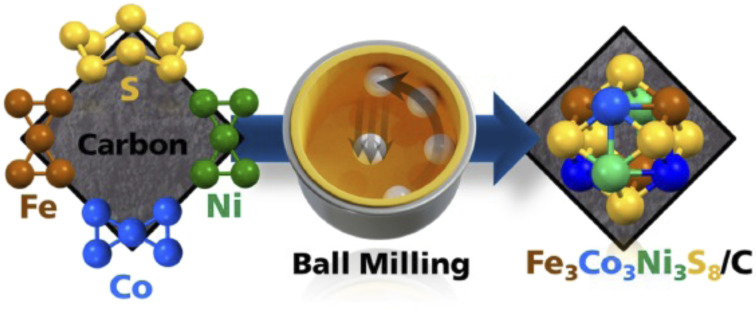

## Introduction

The production of green hydrogen (H_2_) driven by renewable energies is regarded as a key factor in realizing a sustainable hydrogen economy.^1,2^ Currently, especially proton exchange membrane (PEM) electrolyzers rely on scarce noble metals such as Pt to achieve high performance.^3^ Due to the high cost of Pt, researchers have focused over the last few decades on increasing the utilization efficiency (UE) by tuning the size, shape, and face of Pt nanoparticles (NPs).^4^ However, the best UE towards the hydrogen evolution reaction (HER) with Pt NPs has been achieved using supporting materials, which enable maximum surface exposure of noble metals to protons. Nevertheless, Pt remains a critical substance, which necessitates the search for alternative materials. Competing with Pt are inexpensive noble metal-free catalysts and next to transition metal carbides^5^ and pnictides,^6,7^ metal-rich transition metal chalcogenides, in particular of pentlandite (Pn)-type (M_9_X_8_, M = Fe, Co, Ni, X = S, Se), have emerged as promising electrocatalysts.^8^ Their remarkable HER performance has been ascribed to their structural similarity to Fe/Ni-bearing enzymes catalyzing an effective H_2_ production.^9–14^

However, to compete with sophisticated Pt electrodes utilized in industrial PEM electrolysis, both synthesis conditions and the electrochemical performance of Pn-type materials need to be improved. A typical way to improve the HER performance is heterostructuring.^15^ In this process, the active material is chemically or physically bound to another material tuning the electronic structure and increasing active sites, conductivity, and mass transfer. Furthermore, a conductive additive such as carbon is often added during electrode construction as a secondary step.^16^ If these two techniques come together, a high-performance electrode is to be expected. However, approaches to synthesize heterostructured Pn materials thus far yielded low quantities or involved environmentally malign solvothermal methods.^17–23^

Recently, we have reported the mechanochemical synthesis of (Fe,Ni)_9_S_8_ from elemental and sulfidic reaction mixtures.^24^ Mechanochemical reactions are initiated through mechanical force, *e.g.*, during ball milling, and have been shown to be a successful synthesis route for many sulfide and selenide compounds.^25–34^ Compared to traditional synthesis methods, the mechanochemical approach has enabled a cost-effective, scalable, and solvent-free production of pentlandite. Yet, to the best of our knowledge, there is no universal approach yielding a wide range of desired Pn compositions and heterostructured materials.

Herein, we present a mechanochemical “one-fits-all” approach to yield pentlandite/carbon (Fe_3_Co_3_Ni_3_S_8_/C; Pn/C) composites with different carbon materials, which can also be applied to other pentlandite stoichiometries *e.g.*, including selenides. The obtained electrocatalysts are thoroughly characterized and detailed investigations are performed to assess optimal reaction conditions. Furthermore, the electrochemical performance towards the HER of the as-prepared materials is probed followed by utilization in a zero-gap PEM electrolyzer.

## Results and discussion

### Synthesis & characterization

**Fig. 1:**
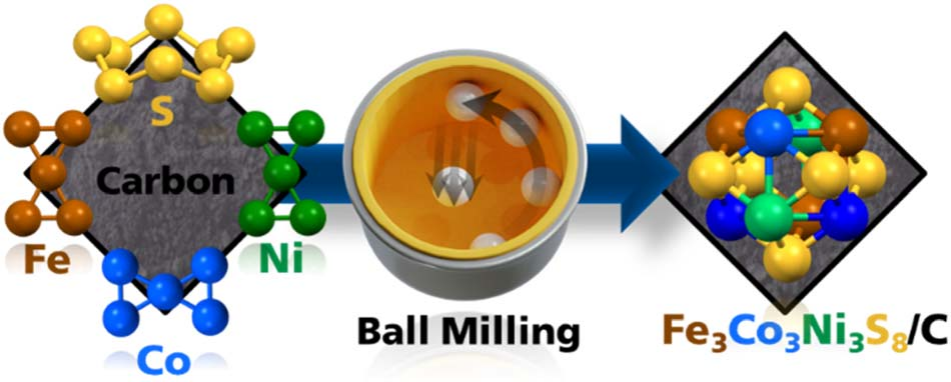
Schematic mechanochemical process for synthesizing heterostructured Pn/C materials.

To synthesize trimetallic pentlandite (Pn) Fe_3_Co_3_Ni_3_S_8_, we followed synthetic protocols recently published by our groups for bimetallic metal sulfides.^24^ We replaced parts of the elemental reaction mixture with carbon black (CB) (10, 25, 50, 75, and 90%) (Fig. 1) as this is a typical additive during electrode assembly and can improve the electrochemical performance. Hereafter, the obtained materials are abbreviated as Pn_*X*CB_, where *X* represents the percentage (wt%) of CB in the sample.

**Fig. 2:**
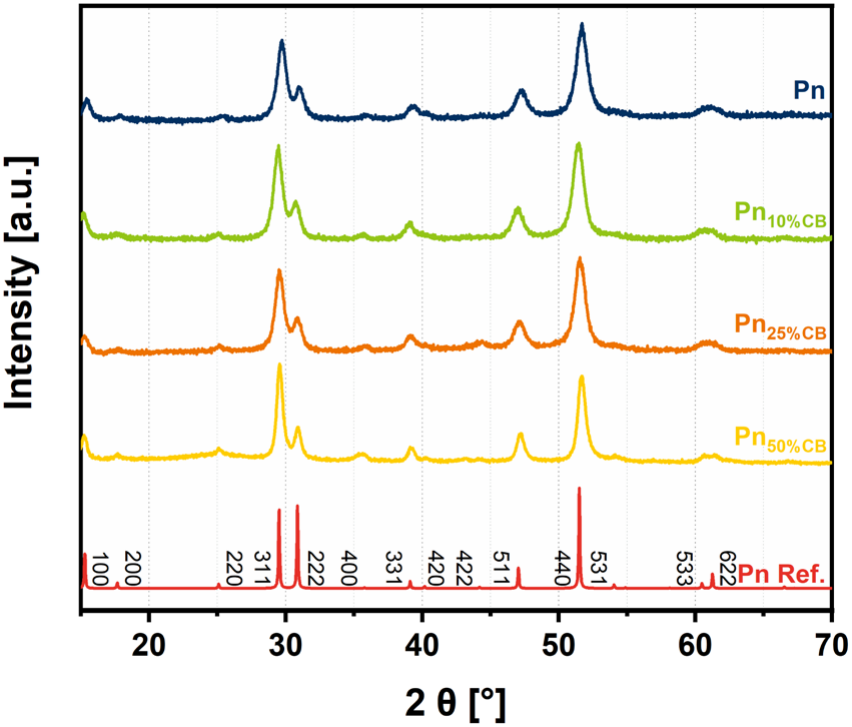
Powder X-ray diffractometry (PXRD) data of the synthesized Pn_*X*CB_ materials displaying powder patterns depending on the carbon amount.

The powder X-ray diffraction (PXRD) patterns of the obtained Pn_*X*CB_ catalysts show a strong dependence on the CB amount. Here, Pn, Pn_10%CB_, and Pn_25%CB_ show similar reflexes in comparison to the pentlandite reference pattern indicating full conversion after 45 min of milling (Fig. 2). Increasing the CB amount to 50% and above leads to an incomplete reaction. Instead, to achieve the desired Pn-phase at higher loadings, the milling time was increased to 6 h for Pn_50%CB_ and Pn_75%CB_ as well as 12 h for Pn_90%CB_, respectively. While a full Pn-conversion for Pn_50%CB_ was observed, Pn_75%CB_ and Pn_90%CB_ did not yield phase pure pentlandite (Fig. S1†). We attribute the hampered Pn-formation to the dilution of Pn with CB, which absorbs the energy from the impact of milling balls. Notably, further elongation of the milling time did not increase the phase purity of Pn_75%CB_ and Pn_90%CB_ (Fig. S1†). Instead, the formation of an amorphous phase was observed, which can be attributed to an extended crystallite shrinkage induced by the milling process. Even though all Pn forms are active in the HER, Pn_75%CB_ and Pn_90%CB_ did not form the desired phase pure Pn_*X*CB_ composites and thus their discussion will be omitted in the following parts. To further understand the reaction progression, gas pressure and temperature were monitored during milling (Fig. S2†). In contrast to our previously reported results, no sharp pressure spike was observed for the given stoichiometries.^24^ Instead, especially samples with higher carbon loading showed steady pressure evolution and temperature increase up to 98 °C. It is known that milling at high frequencies produces higher temperatures inside the vessel, especially when milling at high rotation speeds. The constant pressure increase, however, must be caused by the carbon black (CB) additive, confirmed by reference experiments with pure pentlandite where no drastic pressure increase could be observed at later milling times (Fig. S2†). This behavior is due to a combination of temperature increase and collapse of the CB pore structures followed by the release of adsorbed gas from the CB surface. In contrast to pure carbon black, the pentlandite composite samples show four different regimes, especially pronounced for Pn_50%CB_ samples. The first one is the sharp pressure increase simultaneous to the rising temperature, which is caused by thermal expansion. The second regime thereafter is a slowly rising pressure build-up most likely caused by increasing reaction speed similar to the self-sustaining reaction described before.^24^ Afterwards, the pressure decreases again before rising until the reaction is finished, as distinguished by the full disappearance of the signal at 45° 2*θ*, which can be assigned to a FeNiS_2_ phase. (Fig. S3†). Since Pn_*X*CB_ does not exhibit a typical signal of a self-sustaining reaction, this reaction must proceed through a different reaction mechanism or, more likely, through a similar mechanism, where the reaction is hampered by the dilution with carbon in the reaction mixture. This might lead to the observed smearing of otherwise sharp temperature and pressure responses and could be beneficial for large-scale Pn synthesis that would traditionally be hindered by the highly exothermic character of Pn synthesis.

The stoichiometric composition of the samples was investigated by X-ray fluorescence spectroscopy. In the samples Pn_50%CB_ as well as Pn_10%CB_, the expected elemental ratios of the pentlandite phase were unambiguously confirmed (Table S1†). For Pn_25%CB_, a preferential incorporation of Ni into the otherwise unaffected pentlandite lattice was observed indicating a partially insufficient mixing in the early stage of milling. Importantly, only traces of the milling medium zirconia were detected in all samples. The carbon content was verified by thermogravimetric (TG) analysis (Fig. S4†). Between 450 °C and approx. 850 °C, the oxidation of Pn and carbon occurs, leading to a subsequent CO_2_ and SO_2_ release.^35^ After 850 °C, no mass changes were observed, which was then used to calculate the carbon amount in Pn_10%CB_, Pn_25%CB,_ and Pn_50%CB_ to be 11.9, 28.2, and 50.8%, respectively, confirming the desired carbon amount in each sample.

**Fig. 3:**
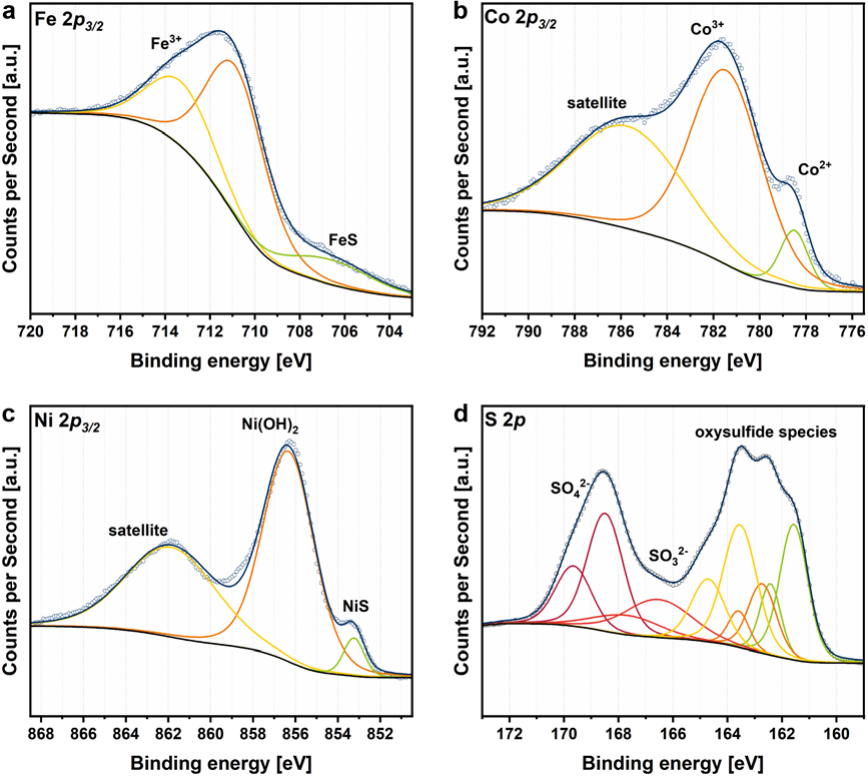
XPS spectra of the Pn_*X*CB_ materials. Spectral deconvolution fits are shown for Pn_10%CB_ as example for (a) Fe 2p_3/2_, (b) Co 2p_3/2_, (c) Ni 2p_3/2_ and (d) S 2p.

**Fig. 4:**
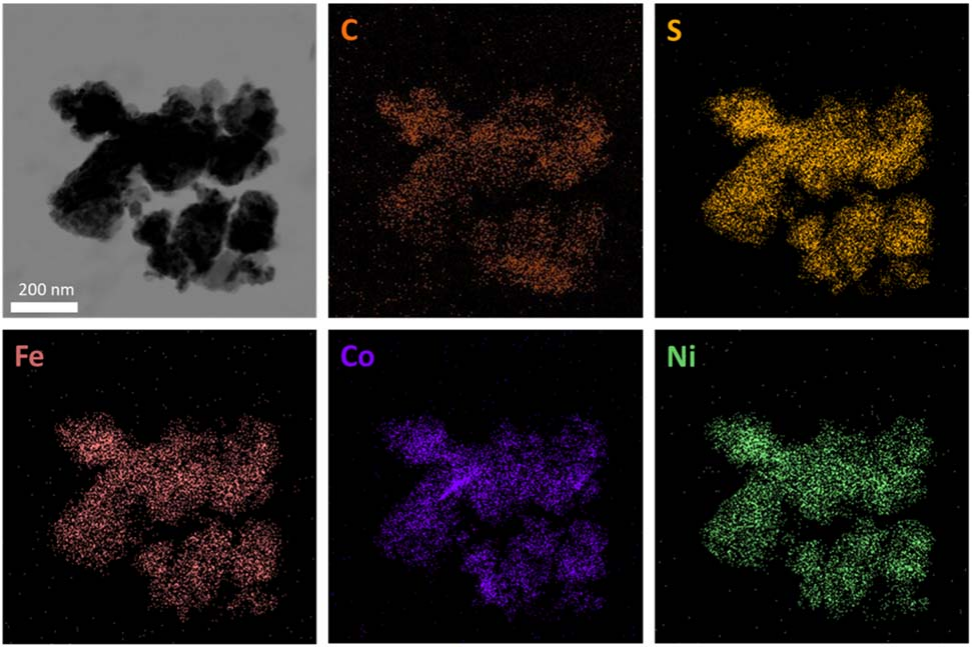
Bright field scanning tunnelling electron microscopy (BF-STEM) image of Pn_25%CB_ including energy dispersive X-ray spectroscopy (EDX) elemental maps displaying a homogeneous distribution of the elements.

X-ray photoelectron spectroscopy (XPS) measurements were performed to assess the composition and oxidation states of the Pn_*X*CB_ materials (Fig. 3 and S5–S7†). All samples display photo-peaks, assignable to the presence of Fe, Co, Ni, and S 2p orbitals (Fig. 3). The deconvolution of the metal 2p_3/2_ regions reveals the occurrence of FeS (707 eV), CoS (779 eV) and NiS (853 eV) as well as Fe-(711 eV), Co-(781 eV) and Ni-(856 eV) oxide species, which is similar to previously published reports.^11^ Notably, the intensity of surface metal sulfide species decreases with increasing carbon amount in the sample. An influence of the carbon amount on the S 2p peaks could also be observed. The increase in the carbon amount is accompanied by a decrease in the S 2p region as well as a shift towards higher binding energies indicating a higher degree of surface sulfur oxidation. Additionally, the higher oxidation degree is represented by an increase of the peak at approx. 168 eV, which can be ascribed to surface SO_4_^2−^ species that form under contact with air. To analyze the distribution and homogeneity of the produced Pn_*X*CB_ composites, transmission electron microscopy (TEM) analysis was performed (Fig. 4 and S8–S10†). Both crystalline and amorphous phases are visible and form irregularly shaped particles. For Pn, a *d*-spacing of 0.25 nm was observed, which corresponds to the (400) plane of the Pn structure (Fig. S8†). At all loadings of CB, the images reveal a homogeneous distribution of carbon over the whole sample. In combination with TEM, energy dispersive X-ray spectroscopy (EDX) was carried out. Here, an equal distribution of the elements Fe, Co, Ni, and S at the pentlandite spots can be observed. TEM revealed particle sizes of around 200 nm. This particle size distribution, however, is not representative due to dispersion-based sample preparation. To remedy this observation, we have performed a particle size distribution analysis by laser diffraction (Fig. S11†). Here, broad particle size distributions of all samples can be observed, with Pn having the smallest particle size with a *D*_90_ value of 5.90 μm. Notably, the particle size distribution of the Pn_10%CB_ and Pn_25%CB_ materials shows a slight shift towards larger particle sizes, which can be attributed to the comparably large C particles added to the reaction mixture. Furthermore, the Pn_50%CB_ material displays the largest particle sizes. Here, the extended milling time, compared to that of Pn_10%CB_ and Pn_25%CB_, may have induced particle agglomeration.

Tuning a material's physicochemical properties towards a specific need can be achieved by altering the materials' composition. To demonstrate the flexibility of the developed process, we have altered the elemental reaction mixture while keeping the CB amount at 25%. By changing the elemental reaction mixture to one containing only Co or Fe and Ni as metal sources, the formation of Co_9_S_8_/C and Fe_4.5_Ni_4.5_S_8_/C was achieved and confirmed by PXRD (Fig. S12†). Furthermore, by substituting one equivalent of S by Se in the trimetallic reaction mixture, we were also able to obtain the sulfoselenide Fe_3_Co_3_Ni_3_S_7_Se/C. In addition to multiple carbon loadings, we were also able to successfully synthesize Pn/C materials with a wide range of different carbon sources such as graphite, reduced graphene oxide (rGO) and carbon nanotubes (CNTs), with each displaying distinct structural and electronic properties (Fig. S13†). Thus, we were able to demonstrate the wide flexibility of the present method, which will ultimately allow for the adjustment of electrode properties using different carbon sources and carbon quantities by altering the hierarchical layering. Conclusively, we compared our process with other strategies to synthesize heterostructured pentlandite materials. The herein described mechanochemical method operates with a low process mass intensity (PMI) and global warming potential (GWP), which describe the effective utilization of reactants and energetic contributions, respectively. Thus, a great environmental benefit, which is often magnitudes better than comparative pathways such as coprecipitation or carbonization methods, is achieved while also operating at the much larger gram-scale (Fig. S14 and Table S2†). Combining this method with the significantly lower price of the necessary resources compared to platinum (Table S3†) puts Pn in a competitive position for large-scale hydrogen production once an abundance of cheap, renewable energy is available.

### Electrochemical HER performance of Pn/C materials

**Fig. 5:**
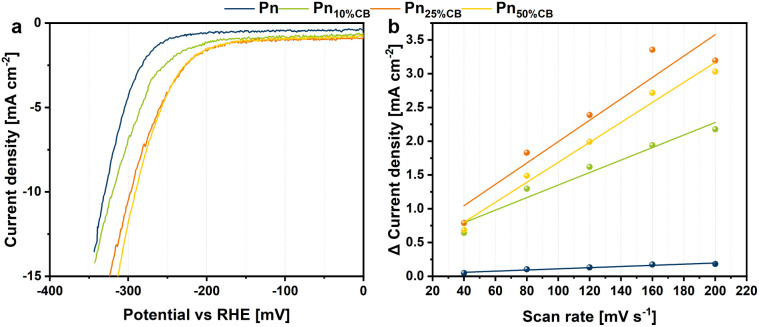
(a) Linear sweep voltammetry (LSV) curves of Pn_*X*CB_ materials at a scan rate of 5 mV s^−1^ and (b) differences of the anodic and cathodic charging current densities plotted against the scan rate for the determination of the double layer capacity (*C*_DL_).

The herein presented mechanochemical procedure enabled a variable synthesis of Pn/C composites with distinct carbon amounts and sources. Furthermore, this study revealed that the ball milling approach can be extended to various elemental compositions and is universally applicable to generate a wide range of carbon-coated and uncoated Pn materials with small particles. To estimate trends in the influence of the carbon amount as an additive for heterostructured Pn materials towards hydrogen evolution, we prepared Pn_*X*CB_ electrodes *via* drop-casting on a classy carbon electrode with a material loading of 0.56 mg cm^−2^. These electrodes served as working electrodes in a three-electrode setup employing an H-type electrolysis cell with a cation exchange membrane in 0.5 M H_2_SO_4_. For the first analysis, the electrochemical activity was determined by linear sweep voltammetry at a scan rate of 5 mV s^−1^ (Fig. 5a). The Pn_*X*CB_ catalyst materials display distinct overpotentials *vs.* RHE at a current density of −10 mA cm^−2^, depending on the catalyst composition. The pentlandite sample with no carbon (Pn) catalyzes the HER at an overpotential of 329 mV. Although the Pn_10%CB_, Pn_25%CB_ and Pn_50%CB_ catalyst materials contain less pentlandite in contrast to Pn, all materials require a lower overpotential of 320, 296 and 290 mV, respectively, thus increasing the utilization efficiency of Pn and clearly showing the importance of the catalyst support. A measurement of the double layer capacitance (*C*_DL_) using cyclic voltammetry shows that the increased activity of Pn_*X*CB_ materials may be derived from an overall increased *C*_DL_ and thus from an increased electrochemical surface area (Fig. 5b).

To further investigate the performance of Pn in conjunction with different carbon materials, we decided to use an industrial relevant zero-gap electrolyzer and prepared catalyst-coated membranes (CCMs) *via* a combined spray coating and hot-pressing method employing the as-prepared Pn_25%C_ and IrO_2_ as cathodic and anodic catalyst materials, respectively. The CCMs were prepared by air-brushing a Pn_25%C_/Nafion ink on one side of a Nafion 212 membrane with a catalyst loading of 3 mg cm^−2^ Pn. The other side of the membrane was coated with an IrO_2_/Nafion ink, which served as the anode. Cross-sectional SEM analyses reveal Pn/C catalyst layer (CL) thicknesses, which are independent of the used carbon material and significantly higher than the IrO_2_ CL (Fig. S15–S18†). XPS analysis of the Pn_25%CB_ CL before electrolysis reveals the absence of the Co^2+^, NiS, and oxysulfide photo-peaks, which can be attributed to superficial oxidation of the catalyst layer induced by the pressing at elevated temperatures (Fig. S19†).

**Fig. 6:**
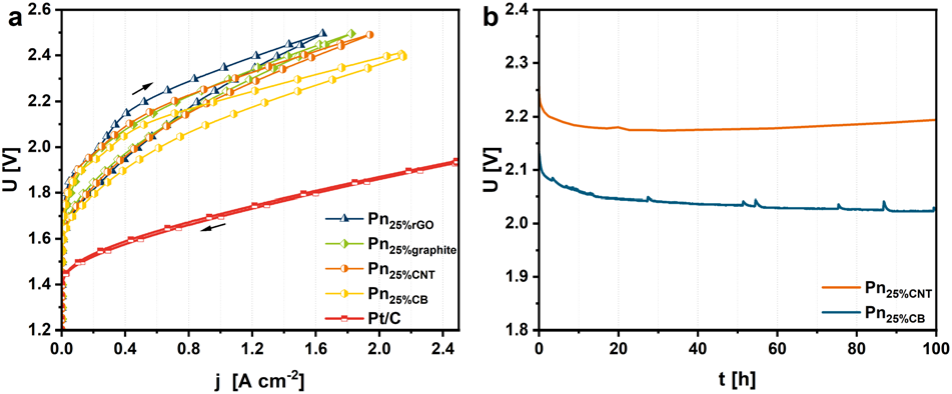
(a) CV curves of Pn_25%C_ and Pt/C in an in-house built PEM electrolyzer recorded with a scan rate of 0.5 mV s^−1^ at 80 °C (left). (b) Chronopotentiometry of Pn_25%CB_ at 80 °C for 100 h at an applied current density of 1 A cm^−2^.

The electrochemical measurements in the zero-gap electrolyzer were then performed supplying both half-cells with only Milli-Q water at 80 °C. In contrast to the H-type cell measurements, no acid is used to feed the system. To condition the CCM and remove superficial oxide species, a conditioning protocol was carried out by conducting chronoamperometric experiments at 1.8 (30 min) and 2.0 V (1 h). Afterward, CV measurements with a scan rate of 0.5 mV s^−1^ were executed to assess the activities of the distinct CCMs. The distinct Pn_25%C_ CCMs display a catalytic activity depending on the utilized carbon material. Here, Pn_25%CB_ has revealed an appreciable cell voltage of 2.12 V at a current density of 1 A cm^−2^ (Fig. 6a). In comparison, the use of CNTs, graphite, and rGO as carbon sources results in overall increased cell potentials of 2.22, 2.23 and 2.26 V, respectively (for a representative GC-MS trace of the hydrogen product stream, see Fig. S28,† and data on a benchmark MEA featuring Pt/C|IrO_2_ for comparison are presented in Fig. S29 and S30†). We attribute the better performance of the Pn_25%CB_ CCM to a high porosity hydrophobic support that does not compete with the active material for water adsorption (Fig. S20–S25†). In detail, the specific surface area for water uptake is the lowest for the Pn_25%CB_ catalyst with a surface area (determined around *p*/*p*_0_ = 0–0.3) of 18 m^2^ g^−1^. In comparison, the Pn_25%graphite_ and Pn_25%rGO_ samples show increased surface areas of 63 and 91 m^2^ g^−1^, respectively. Yet, Pn_25%CNT_ revealed a specific surface for water adsorption of 0 m^2^ g^−1^, indicating that a fully hydrophobic surface is not beneficial for the process. In addition, we determined the water uptake at *p*/*p*_0_ = 0.975 to gain insights into the material's pore volumes. Herein, Pn_25%graphite_ and Pn_25%CB_ showed the lowest water uptake capacity of 0.08 and 0.09 cm^3^ g^−1^, respectively, indicating the smallest pore volume. In comparison, Pn_25%rGO_ shows the highest water uptake of 0.21 cm^3^ g^−1^, which is in accordance with the high specific surface area for water uptake. Although Pn_25%CNT_ has a specific surface area for water uptake of 0, a water uptake of 0.15 cm^3^ g^−1^ was observed. Thus, seemingly a low water uptake within the pores as well as a low specific surface for water is beneficial for the overall HER performance.

Notably, in contrast to the Pt/C|IrO_2_-benchmark (Fig. S29†), the CV measurements of the Pn_25%C_ catalysts display a slight hysteresis, which decreases with higher cell voltages. For example, the hysteresis of Pn_25%CB_ includes an 80 mV span at 1 A cm^−2^ from 2.20 V for the forward to 2.12 V for the backward scan. This behavior yet remains elusive and is currently subject to further investigations. Furthermore, we have performed chronopotentiometry measurements for Pn_25%CB_ and Pn_25%CNT_ at 1 A cm^−2^, which showed a stable performance of the MEAs for at least 100 h at the high current densities (Fig. 6b). As anticipated from the CV measurements as well as the water sorption measurements, Pn_25%CB_ catalyzes the electrolysis with improved performance displaying lower cell voltages of up to 150 mV after 100 h in contrast to Pn_25%CNT_.

Altogether, we showed the generation of green hydrogen in conjunction with an industrial PEM electrolyzer at elevated current densities of 1 A cm^−2^ for 100 h using the trimetallic pentlandite catalyst Fe_3_Co_3_Ni_3_S_8_. By tuning the hierarchical layers in the CCMs using different carbon sources we were able to increase the performance of PGM-free catalyst materials and thus emphasize the importance of the catalyst support. We are convinced that further optimization of the catalyst ink formulation as well as the synthetic parameters will significantly improve the catalyst performance of the herein introduced Pn/C materials.

## Conclusions

We herein demonstrate the rapid and eco-friendly gram-scaled synthesis of heterostructured trimetallic pentlandite/carbon composites (Fe_3_Co_3_Ni_3_S_8_/C, Pn/C). This is the first report demonstrating a “one-fits-all” method to yield a multitude of different pentlandite compositions as well as heterostructured Pn/C composites from elemental reaction mixtures, which allows for the further tuning of the physicochemical properties of this material class. In detail, phase-pure Pn/C catalyst materials with a variable carbon amount of up to 50% were obtained through a mechanochemical route utilizing simply the pure elements. These synthetic protocols serve as a blueprint to enable the synthesis of mono- and bi-metallic as well as sulfoselenide pentlandite materials with low environmental impact and such protocols can be easily adapted to other carbon sources (*e.g.* carbon nanotubes (CNTs) or reduced graphene oxide (rGO)).

Furthermore, we utilized the Pn/C materials as electrocatalysts for the hydrogen evolution reaction (HER) and showed enhanced HER activity with an increasing carbon amount attributed to an increased overall electrochemical surface area. The Pn_25%C_ materials revealed high durability as cathodic HER catalysts in a zero-gap PEM cell at 1 A cm^−2^ for at least 100 h.

While the performance of Pt cannot be reached yet, we herein demonstrate the significant influence of the catalyst support material on the overall performance of noble-metal-free electrocatalysts and show the need to control the material's wettability. In summary, the electrode assembly is as important as the catalyst used. The combination of active material conception with electrode formulation makes this route especially interesting for the industry.

## Experimental

### Chemicals

All utilized chemicals were provided by commercial vendors and were used without purification. A detailed list can be found in Table S5.†

### Synthesis of Fe_3_Co_3_Ni_3_S_8_

The Fe_3_Co_3_Ni_3_S_8_ electrocatalyst was synthesized according to a method recently published by our group.^24^ The mechanochemical synthesis was realized using a Fritsch Pulverisette 7 premium line with ZrO_2_ milling containers (V = 20 mL) and ZrO_2_ milling balls (*d* = 2 mm and *m* = 24 g). A reaction mixture (*m* = 1 g) composed of stoichiometric amounts of Fe, Co, Ni, and S was prepared inside a glovebox to ensure an inert argon atmosphere inside the milling containers. The mechanochemical reaction was performed at a constant rotation speed of 1100 rpm for 45 min.

### Synthesis of Fe_3_Co_3_Ni_3_S_8_/C materials

The synthesis of Fe_3_Co_3_Ni_3_S_8_/C was realized analogously to the synthesis of Fe_3_Co_3_Ni_3_S_8_. Instead, distinct portions of 10, 25, or 50 wt% of the metal sulfide reaction mixture were replaced by the carbon source. The mechanochemical syntheses were performed at a constant rotation speed of 1100 rpm with variable milling durations depending on the amount and the choice of the selected carbon source. A list of milling durations is provided in Table S6.†

### Physical characterization

The phase identification of the catalyst materials was carried out using a Bruker Phaser D2 powder diffractometer equipped with a Cu Kα radiation source (*λ* = 1.5406 Å) at 30 kV and 10 mA.

The particle sizes of the synthesized electrocatalysts were determined using a Shimadzu SALD-2300 laser diffraction particle size analyzer equipped with a SALD-BC23 batch cell. The respective samples were prepared by dispersing approx. 10 mg of the catalyst material for 1 min in 1 mL isopropyl alcohol using an ultra-sonication bath. Subsequently, a portion of the dispersion was added to the isopropyl alcohol-filled batch cell. The obtained particle sizes were calculated using the Fraunhofer approximation.

The X-ray fluorescence (XRF) spectra were obtained by using a Vanta™ Handheld XRF Analyzer from OLYMPUS with a 40 keV rhodium X-ray tube and a silicon PIN-detector.

X-ray photoelectron spectroscopy (XPS) was performed on a Nexsa G2 from Thermo Fisher Scientific equipped with an Al Kα radiation source operated at 12 keV and a Quartz 0.25 m Rowland circle monochromator. The device is equipped with bipolar analyzer electronics (ISS) with an energy range between 0 and 1500 eV and pass energy between 1 and 400 eV. Furthermore, reflected electron energy loss spectroscopy (REELS) and charge compensation with an electron beam energy between 0 and 1000 eV and UV photoelectron spectroscopy were performed.

High-resolution transmission electron microscopy (HRTEM) was carried out with a JEM 2800 microscope from JEOL. It was equipped with a Schottky-type emission source working at 200 kV and a Gatan OneView camera (4k × 4k, 25 FPS) to obtain images with a resolution of 0.09 nm. Energy dispersive X-ray analysis (EDX) elemental mapping was performed using double silicon drift detectors (SDDs), with a solid angle of 0.98 steradians with a detection area of 100 mm^2^.

Thermogravimetry (TG) measurements were performed on a Netzsch STA 449-F3. Approx. 50 mg of the sample was placed in a corundum crucible and heated from 27 to 1000 °C at a heating rate of 10 k min^−1^. A nitrogen/air (80 : 20) purging gas flow rate of 50 mL and a nitrogen protecting flow rate of 20 mL were applied. The temperature was calibrated against high-purity Au, Ag, In, and Zn standards.

Scanning electron microscopy (SEM) images for cross-sectional analyses were recorded using a ZEISS Gemini2 Merlin HR-FESEM coupled with an OXFORD Aztec Energy X-ray microanalysis system for energy dispersive X-ray spectroscopy (EDX). The SEM images were recorded using an acceleration voltage of 20 kV.

Water physisorption measurements were performed at 298 K by using an Autosorb-IQ-C-XR (Quantachrome Instruments). Specific surface areas (*S*_BET_) were calculated by using the BET equation in a relative pressure range that fits the consistency criteria proposed by Rouquerol *et al.*^36^ Total water uptakes were measured at *p*/*p*_0_ = 0.95. Before physisorption experiments, all samples were activated at 423 K for 24 h under vacuum.

### Electrochemical measurements

The Pn/CB electrocatalysts were investigated as drop-casted materials on a glassy carbon (GC) rod electrode. For this purpose, 13.19 mg of the catalyst material was dispersed in a mixture composed of 0.49 mL ethylene glycol, 0.49 mL isopropyl alcohol, and 0.02 mL Nafion (5% in aliphatic alcohols) using an ultra-sonication bath for 30 min. Then, 3 μL of the catalyst ink was applied on a GC electrode (*d* = 3 mm and 0.56 mg cm^−2^ catalyst), which was dried in an oven. Before drop casting, the GC electrode was polished using Al_2_O_3_ pastes with grain sizes of 0.30 and 0.05 μm for 3 min each, followed by ultra-sonication in Milli-Q water for 5 min.

Electrochemical measurements were performed in a three-electrode setup employing the catalyst-modified GC working, a Pt mesh counter, and an Ag/AgCl (sat. KCl) reference electrode in 0.5 M H_2_SO_4_. The working and counter electrodes were separated by utilizing an H-type electrolysis cell with both half cells being separated by a cation exchange membrane (FUMASEP F-10120PK). Electrochemical measurements were conducted with the help of a GAMRY Reference 600 or Reference 600+ and the measured potentials were converted to the RHE reference according to:

1 *E*_RHE_ = *E*_measured_ + *E*_ref_ + 0.059 × pH

where *E*_RHE_ is the potential of the reversible hydrogen electrode, *E*_measured_ is the measured potential, and *E*_ref_ is the potential of the reference electrode.

For the HER experiments, the material was first electrochemically conditioned through cyclic voltammetry (CV) between 0 and −0.3 V *vs.* RHE at 100 mV s^−1^ to remove oxide species on the surface of the catalyst materials until a stable voltammogram was obtained. The investigation of the electrochemical surface area (ECSA) was realized through CV measurements between −0.1 and −0.2 V *vs.* RHE at scan rates of 40, 80, 120, 160, and 200 mV s^−1^. Changes in the electrochemical activity were monitored *via* linear sweep voltammetry between 0 and −0.35 V *vs.* RHE at a scan rate of 5 mV s^−1^.

### Measurements in a zero-gap PEM electrolyzer

The preparation of the catalyst ink was performed by dispersing a mixture of 0.4 g of Pn_25%C_, 7.8 g Milli-Q water, and 1 g Nafion solution D2020 (20%, ion power) which was diluted to 40 mL with isopropyl alcohol for 15 min using an ultrasonic bath followed by stirring for 12 h. The preparation of the Pt/C (40%, Quintech) catalyst reference ink involved mixing 0.4 g catalyst with 1 g Nafion solution D2020 (ion power) (20%) and 7.8 g Milli-Q, which was subsequently diluted to 30 mL with isopropyl alcohol. For the anode, 0.4 g of IrO_2_ were mixed with 0.353 g Nafion solution and diluted similarly to the Pt/C ink. The Pt/C and the IrO_2_ ink were dispersed with an Ultra-Turrax T18D at 13 000 rpm for 1 min and were then stirred continuously.

For the membrane coating, a hand-held Iwata Eclipse with compressed nitrogen gas was used. A Nafion 212 (Fuel Cell Store) membrane was placed on a hot plate at 80 °C and first, the IrO_2_ ink was sprayed followed by the Pn_25%C_ or the Pt/C ink until catalyst loadings of 1 mg cm^−2^ Ir, 3 mg cm^−2^ Pn_25%C_ and 1 mg cm^−2^ Pt were achieved. Subsequently, the membrane was hot pressed at 135 °C with 10 bar (0.1 kN cm^−2^) for 90 s with a Polystat 300S hot press. The membrane was then placed in Milli-Q water for 12 h followed by installation into an in-house built PEM electrolyzer. The single cell electrolyzer contains electrodes of an area of 12.57 cm^−2^ and was tightened with a torque of 5 Nm. Furthermore, PTFE gaskets, Ti flow fields, and copper plates as the anodic collector and cathodic collector feed were employed. Milli-Q water at 80 °C was circulated through both half-cells at a flow rate of 50 mL min^−1^ during the measurements.

The electrochemical measurements were performed with a Gamry Reference 3000 including a Reference 30k Booster. Electrochemical conditioning for removing superficial oxide species on the cathode of the membrane electrode assembly (MEA) was performed by applying 1.8 V for 30 min and 2.0 V for 60 min. Afterward, cyclic voltammetry measurements were performed with a scan rate of 0.5 mV s^−1^. Electrolysis at 1 A cm^−2^ for 100 h was performed using Pn_25%CB_.

## Data availability

Data supporting this study is available in the ESI† and further details are available from the authors on reasonable request.

## Author contributions

DT and TR performed the synthesis and basic electrochemical characterisations. Both also prepared the first draft of the manuscript. LM and PB were responsible for MEA fabrication and testing in zero gap cells. SG performed and evaulated the XPS experiments. DS, LB and UPA supervised the study. All authors were involved in the manuscript preparation and data evaluation.

## Conflicts of interest

There are no conflicts to declare.

## Supplementary Material

SC-014-D3SC04542K-s001

## References

[cit1] Chu S., Majumdar A. (2012). Nature.

[cit2] Siegmund D., Metz S., Peinecke V., Warner T. E., Cremers C., Grevé A., Smolinka T., Segets D., Apfel U.-P. (2021). JACS Au.

[cit3] Greeley J., Markovic N. M. (2012). Energy Environ. Sci..

[cit4] Zhu J., Hu L., Zhao P., Lee L. Y. S., Wong K.-Y. (2020). Chem. Rev..

[cit5] Michalsky R., Zhang Y.-J., Peterson A. A. (2014). ACS Catal..

[cit6] Ge Z., Fu B., Zhao J., Li X., Ma B., Chen Y. (2020). J. Mater. Sci..

[cit7] Theerthagiri J., Lee S. J., Murthy A. P., Madhavan J., Choi M. Y. (2020). Curr. Opin. Solid State Mater. Sci..

[cit8] Siegmund D., Blanc N., Smialkowski M., Tschulik K., Apfel U.-P. (2020). ChemElectroChem.

[cit9] Konkena B., Junge Puring K., Sinev I., Piontek S., Khavryuchenko O., Dürholt J. P., Schmid R., Tüysüz H., Muhler M., Schuhmann W., Apfel U.-P. (2016). Nat. Commun..

[cit10] Möller F., Piontek S., Miller R. G., Apfel U.-P. (2018). Chem.–Eur. J..

[cit11] Piontek S., Andronescu C., Zaichenko A., Konkena B., Junge Puring K., Marler B., Antoni H., Sinev I., Muhler M., Mollenhauer D., Roldan Cuenya B., Schuhmann W., Apfel U.-P. (2018). ACS Catal..

[cit12] Smialkowski M., Tetzlaff D., Hensgen L., Siegmund D., Apfel U.-P. (2021). Chin. J. Catal..

[cit13] Tang Y., Yang H., Sun J., Xia M., Guo W., Yu L., Yan J., Zheng J., Chang L., Gao F. (2018). Nanoscale.

[cit14] Smialkowski M., Siegmund D., Pellumbi K., Hensgen L., Antoni H., Muhler M., Apfel U.-P. (2019). Chem. Commun..

[cit15] Zhao G., Rui K., Dou S. X., Sun W. (2018). Adv. Funct. Mater..

[cit16] Hughes J. P., Clipsham J., Chavushoglu H., Rowley-Neale S. J., Banks C. E. (2021). Renewable Sustainable Energy Rev..

[cit17] Cao D., Kang W., Huang Z., Li H., Yang M., Li J., Gao Y., Wang Y., Ma P., Sun D. (2019). Electrochim. Acta.

[cit18] Wang F., Li K., Li J., Wolf L. M., Liu K., Zhang H. (2019). Nanoscale.

[cit19] Al-Mamun M., Wang Y., Liu P., Zhong Y. L., Yin H., Su X., Zhang H., Yang H., Wang D., Tang Z., Zhao H. (2016). J. Mater. Chem. A.

[cit20] Bezverkhyy I., Afanasiev P., Danot M. (2004). J. Phys. Chem. B.

[cit21] Kim Y., Karuppannan M., Lee D., Bae H. E., Luong Q. T., Kang S. Y., Sung Y.-E., Cho Y.-H., Kwon O. J. (2021). Int. J. Energy Res..

[cit22] Roffey A., Hollingsworth N., Hogarth G. (2019). Nanoscale Adv.

[cit23] Wang F., Li K., Li J., Wolf L. M., Liu K., Zhang H. (2019). Nanoscale.

[cit24] Tetzlaff D., Pellumbi K., Baier D. M., Hoof L., Shastry Barkur H., Smialkowski M., Amin H. M. A., Grätz S., Siegmund D., Borchardt L., Apfel U.-P. (2020). Chem. Sci..

[cit25] Baláž P., Dutková E., Baláž M., Džunda R., Navrátil J., Knížek K., Levinský P., Hejtmánek J. (2021). ChemistryOpen.

[cit26] Andersen J., Mack J. (2018). Green Chem..

[cit27] Hlova I. Z., Singh P., Malynych S. Z., Gamernyk R. V., Dolotko O., Pecharsky V. K., Johnson D. D., Arroyave R., Pathak A. K., Balema V. P. (2021). Nanoscale Adv.

[cit28] Mottillo C., Friščić T. (2017). Molecules.

[cit29] Rounaghi S. A., Eshghi H., Scudino S., Esmaeili E., Kiani-Rashid A.-R., Eckert J. (2019). Phys. Chem. Chem. Phys..

[cit30] Achimovičová M., Baláž M., Girman V., Kurimský J., Briančin J., Dutková E., Gáborová K. (2020). Nanomaterials.

[cit31] Baláž P., Achimovičová M., Baláž M., Chen K., Dobrozhan O., Guilmeau E., Hejtmánek J., Knížek K., Kubíčková L., Levinský P., Puchý V., Reece M. J., Varga P., Zhang R. (2021). ACS Sustainable Chem. Eng..

[cit32] Shalabayev Z., Baláž M., Khan N., Nurlan Y., Augustyniak A., Daneu N., Tatykayev B., Dutková E., Burashev G., Casas-Luna M., Džunda R., Bureš R., Čelko L., Ilin A., Burkitbayev M. (2022). Nanomaterials.

[cit33] Baláž M., Tkáčiková L., Stahorský M., Casas-Luna M., Dutková E., Čelko L., Kováčová M., Achimovičová M., Baláž P. (2022). ACS Omega.

[cit34] Gáborová K., Achimovičová M., Hegedüs M., Girman V., Kaňuchová M., Dutková E. (2022). Front. Chem. Sci. Eng..

[cit35] Dunn J. G., Kelly C. E. (1980). J. Therm. Anal..

[cit36] Rouquerol J. , Llewellyn P., and Rouquerol F., in Characterization of porous solids VII, ed. P. L. Llewellyn, Elsevier, Amsterdam, Boston, 1st edn., 2007, pp. 49–56

