# Supplementary material for: Mechanochemical one-pot synthesis of heterostructured pentlandite-carbon composites for the hydrogen evolution reaction
Source: Chem Sci. 2023 Sep 29;14(42):11790–7. doi: 10.1039/d3sc04542k (PMC10619543; doi:10.1039/d3sc04542k)
Supplement: SC-014-D3SC04542K-s001 [file SC-014-D3SC04542K-s001.pdf]

Electronic Supplementary Material (ESI) for Chemical Science.
This journal is © The Royal Society of Chemistry 2023

Mechanochemical One-Pot Synthesis of heterostructured Pentlandite-Carbon Composites for the Hydrogen Evolution Reaction

SUPPORTING INFORMATION

David Tetzlaff,‡[ab] Tilo Rensch,‡[b] Leonard Messing,[a] Petra Banke,[a] Sven Grätz,[b] Daniel Siegmund,[ab] Lars Borchardt*[b] and Ulf-Peter Apfel**[ab]

a) Fraunhofer UMSICHT, Osterfelder Straße 3, DE-46047 Oberhausen, Germany

b) Ruhr University Bochum, Inorganic Chemistry I, Universitätsstraße 150, DE-44780 Bochum, Germany

‡ These authors contributed equally to this work.

Corresponding author.

**Email: lars.borchardt@rub.de*

***Email: ulf.apfel@rub.de or ulf.apfel@umsicht.fraunhofer.de*

Contents

Powder X-ray diffraction (PXRD) analysis of the synthesized amorphous **Pn$_{XCB}$** materials .. 3

EASY-GTM gas pressure and temperature measurements during the synthesis of **Pn/CB** materials and time-resolved PXRD-Analysis of **Pn$_{50\%CB}$** 4

Calculation of maximal gas evolution from carbon black 5

X-ray fluorescence (XRF) analysis of **Pn$_{XCB}$** materials 6

Thermogravimetry (TG) measurements of **Pn$_{XCB}$** materials 7

X-ray photoelectron spectroscopy (XPS) spectra of the **Pn$_{XCB}$** materials 8

Transmission electron microscopy (TEM) images coupled with energy dispersive X-ray emission spectroscopy (EDX) mappings of **Pn** 10

Transmission electron microscopy (TEM) images coupled with energy dispersive X-ray emission spectroscopy (EDX) mappings of **Pn$_{10\%CB}$** 11

Transmission electron microscopy (TEM) images coupled with energy dispersive X-ray emission spectroscopy (EDX) mappings of **Pn$_{50\%CB}$** 12

Laser diffraction measurements of the synthesized **Pn$_{XCB}$** materials 13

Process Flexibility 14

Environmental Analysis 15

Cost analysis 17

Cross sectional SEM image of a catalyst coated membrane using **Pn$_{25\%CB}$** as cathodic catalyst 18

Cross sectional SEM image of a catalyst coated membrane using **Pn$_{25\%graphite}$** as cathodic catalyst 19

Cross sectional SEM image of a catalyst coated membrane using **Pn$_{25\%CNT}$** as cathodic catalyst 20

Cross sectional SEM image of a catalyst coated membrane using **Pn$_{25\%rGO}$** as cathodic catalyst 21

Table of catalyst layer thicknesses of the prepared cathodes determined via SEM........... 22

X-ray photoelectron spectroscopy (XPS) spectra of the **Pn$_{25\%CB}$** CCM catalyst layer. 23

Water vapor sorption analysis of **Pn$_{25\%CB}$** 24

Water vapor sorption analysis of **Pn$_{25\%graphite}$** 25

Water vapor sorption analysis of **Pn$_{25\%CNT}$** 26

Water vapor sorption analysis of **Pn$_{25\%rGO}$** 27

Electrochemical MEA data 28

Materials 31

Milling durations for obtaining distinct **Pn/C** materials.. 32

References 33

Powder X-ray diffraction (PXRD) analysis of the synthesized amorphous **Pn$_{XCB}$** materials

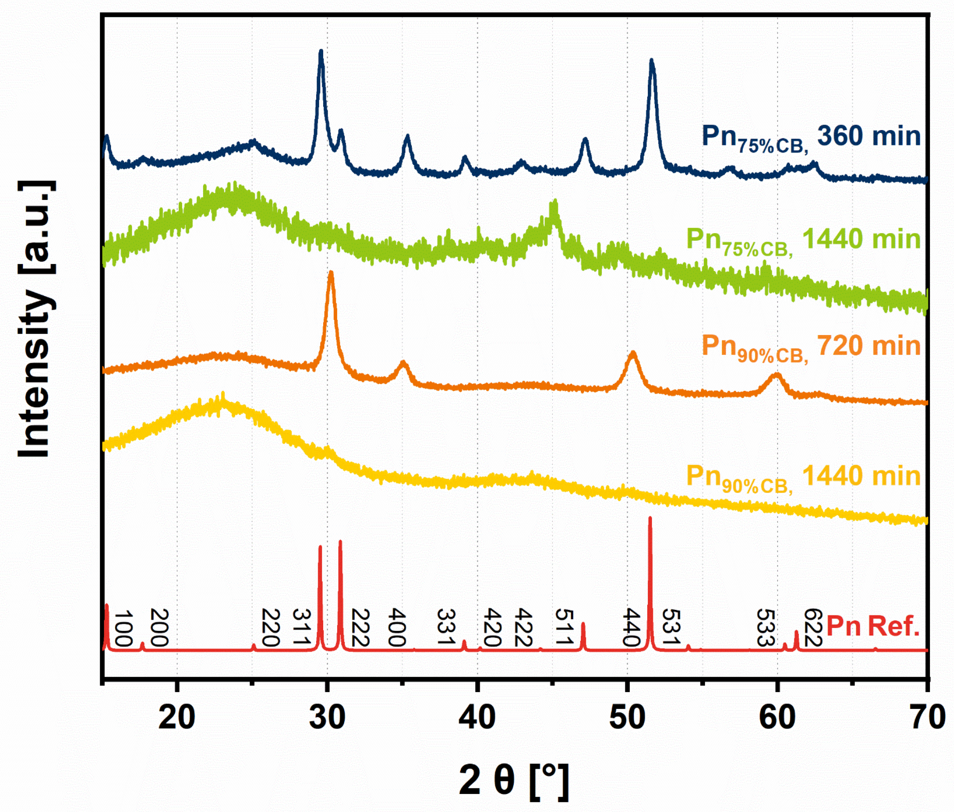

Fig. S1: Powder X-ray analysis of the synthesized **Pn$_{75\%CB}$** and **Pn$_{90\%CB}$** materials displaying amorphous phases.

EASY-GTM gas pressure and temperature measurements during the synthesis of **Pn/CB** materials and time-resolved PXRD-Analysis of **Pn$_{50\%CB}$**

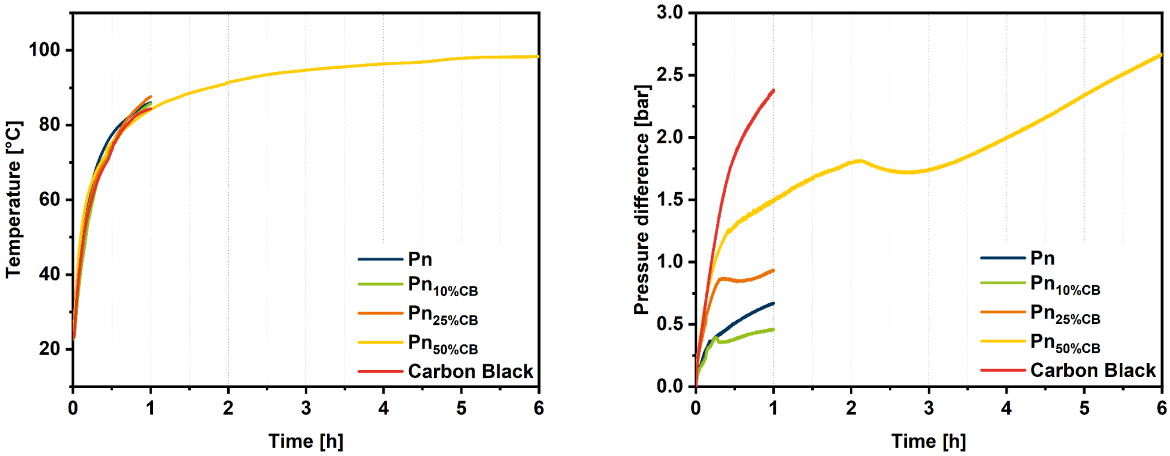

Fig. S2: Temperature (left) and pressure (right) changes during the synthesis of **Pn$_{XCB}$** materials.

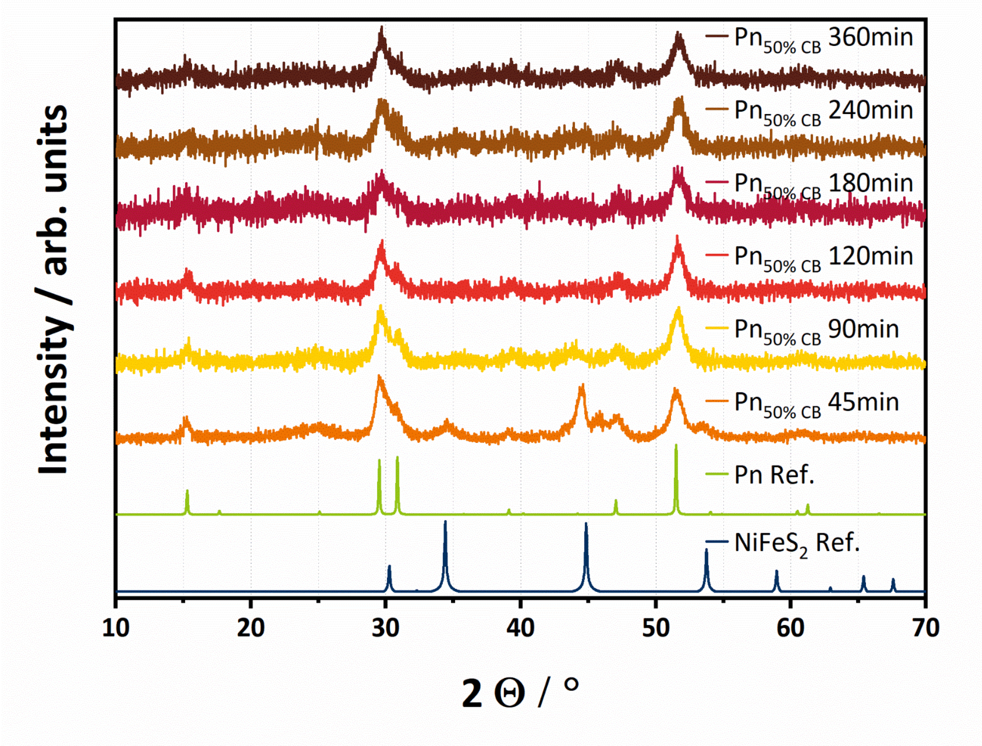

Fig. S3: Time-resolved Powder X-ray analysis of the synthesized **Pn$_{50\%CB}$** displaying the structural changes from $NiFeS_2$ to the pentlandite phase.

Calculation of maximal gas evolution from carbon black

The assumptions made are:

1. Monolayer adsorption of only N_2 at the carbon BET surface
   a. Monolayer thickness: $h_{monolayer}$ 0.354 nm.[1]
   b. Area per molecule: A_{N2} 0.162 nm^2.[2]
2. Ideal gas behavior
3. No volume occupied in the beaker by carbon
4. 23 g of ZrO_2 balls in 20 mL beaker with constant volume totaling in 16 cm^3 free volume.
5. Two separate state changes:
   a. Isochoric release of adsorbed species and pore volume
   b. Isochoric temperature increasec

Nitrogen physisorption data for BET surface area and pore volume of the used carbon black are published by Grützmacher et al.[3]. Start (T1) and end temperatures (T2) were taken as measured (Fig. S2) for 1 g of carbon black material.

$$N_{ads,N_2} = \frac{A_{BET}}{A_{N_2}} \cdot m_C = \frac{254\ {m^2}/{g}}{162 \cdot 10^{-21} m^2} \cdot 1g = 1.567 \cdot 10^{21} \tag{1}$$

$$n_{ads,N_2} = \frac{N_{ads,N_2}}{N_A} = \frac{1.567 \cdot 10^{21}}{6.022 \cdot 10^{23}\ {1}/{mol}} = 2.60 \cdot 10^{-3} mol \tag{2}$$

$$V_{eff.pore} = V_{pore} \cdot m_C - V_{ads,N_2} = V_{pore} \cdot m_C - A_{N_2} \cdot h_{monolayer} \cdot N_{ads,N_2} \tag{3}$$

$$V_{eff.pore} = 1.74\ {cm^3}/{g} \cdot 1g - 162 \cdot 10^{-17} cm^2 \cdot 354 \cdot 10^{-10} cm \cdot 2.60 \cdot 10^{-3} mol = 1.65 cm^3 \tag{4}$$

$$n_{C,N_2} = \frac{V_{eff.pore}}{V_M} + n_{ads,N_2} = \frac{1.65 cm^3}{22.4 \cdot 10^3\ {cm^3}/{mol}} + 2.60 \cdot 10^{-3} mol = 2.67 \cdot 10^{-3} mol \tag{5}$$

$$p_1 = \frac{n_{C,N_2} RT}{V} = \frac{2.67 \cdot 10^{-3} mol \cdot 8.314\ {J}/{Kmol} \cdot 296.25}{16 \cdot 10^{-6} m^3} = 409.477 kPa \tag{6}$$

$$p_2 = \frac{p_2 \cdot T_2}{T_1} = \frac{409.477 kPa \cdot 357.95 K}{296.25 K} = 494.758 kPa \approx \underline{4.95 bar} \tag{7}$$

This calculation provides an estimate pressure increase of 4.95 bar for total pore collapse under mechanochemical conditions. The measured pressure increase of 2.5 bar (Fig. S2) would thus correspond to a collapse of half of the pore structure with desorption of gas at 50 % of available surface area (1.3 mmol).

X-ray fluorescence (XRF) analysis of $\mathbf{Pn_{XCB}}$ materials

Table S1: X-ray fluorescence (XRF) analysis of $\mathbf{Pn_{XCB}}$ materials for the determination of the elemental composition.

Sample	Fe [m%]	Co [m%]	Ni [m%]	Zr [m%]	Fe calc. [m%]	Co calc. [m%]	Ni calc. [m%]
10 % CB	10,9	10,3	13,0	0,065	10,8	11,4	11,3
25 % CB	24,1	30,5	44,9	0,196	19,4	20,5	20,4
50 % CB	11,8	11,1	15,0	0,309	10,8	11,4	11,3
Vulcan Carbon Black	<LOD	<LOD	<LOD	0,026	0,0	0,0	0,0

Thermogravimetry (TG) measurements of Pn_{XCB} materials

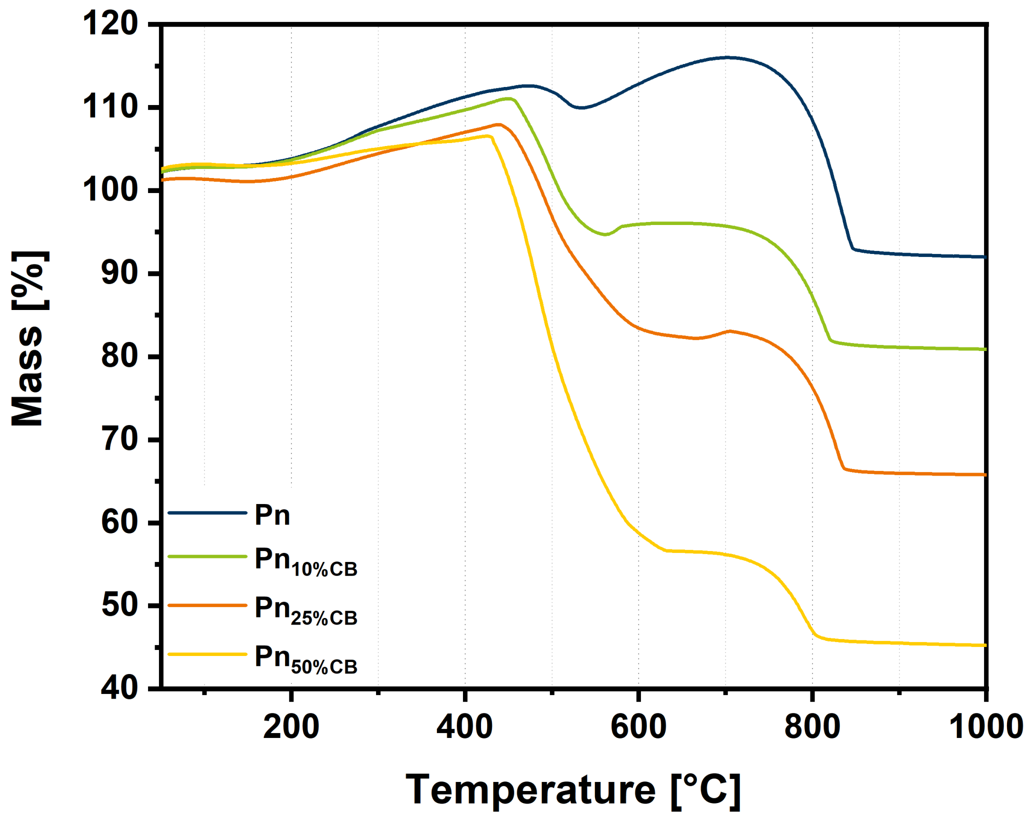

Fig. S4: Thermogravimetric measurements of the synthesized **Pn_{XCB}** materials for the determination of the carbon amount in the samples.

X-ray photoelectron spectroscopy (XPS) spectra of the $\mathbf{Pn_{XCB}}$ materials

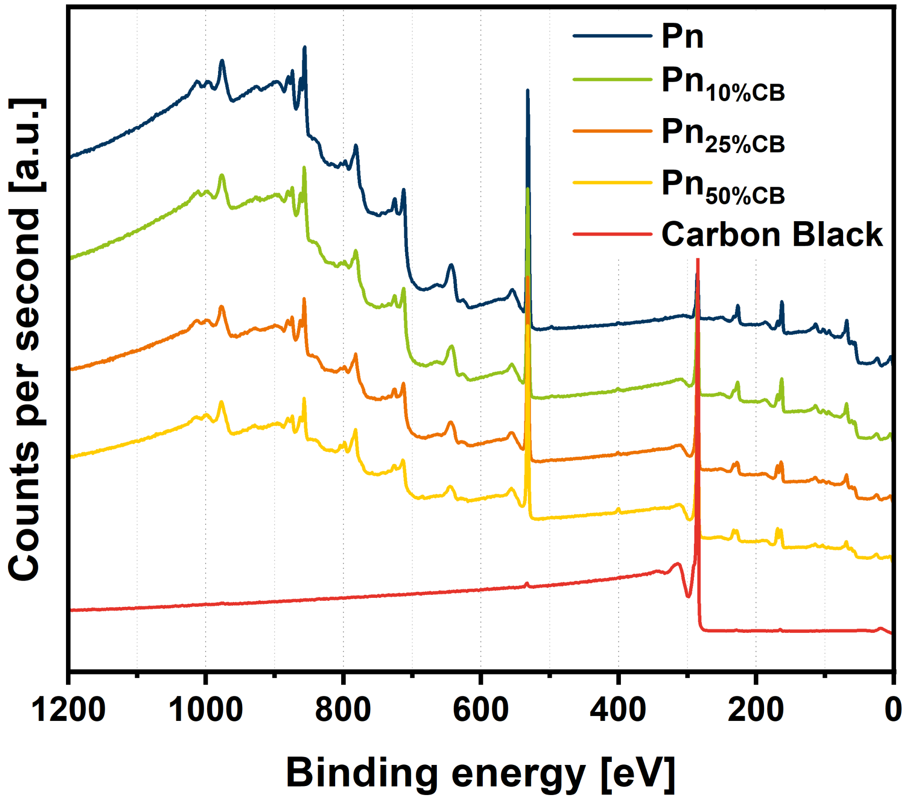

Fig. S5: X-ray photoelectron spectroscopy (XPS) survey spectra of the $\mathbf{Pn_{XCB}}$ materials including carbon black as reference.

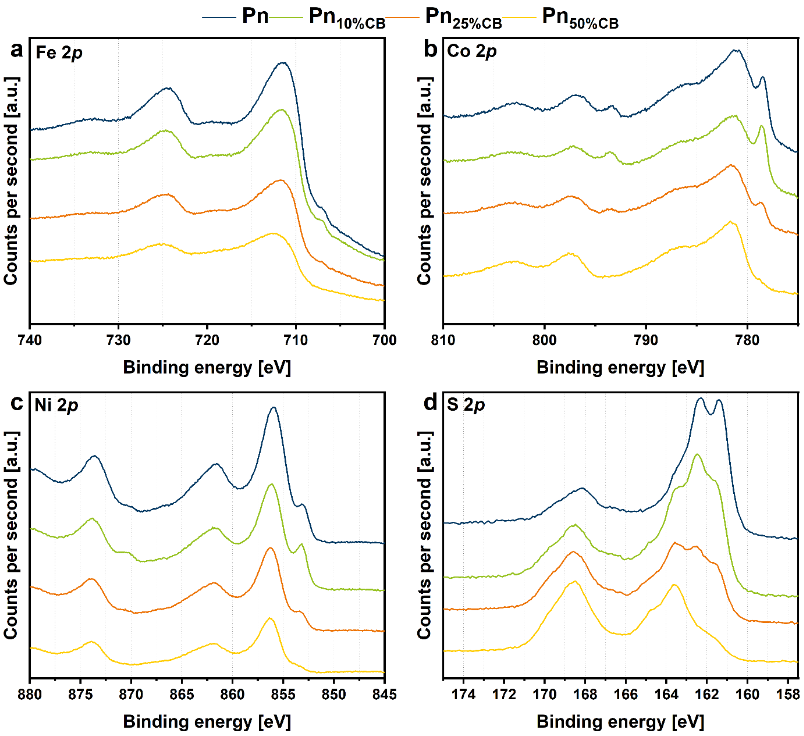

Fig. S6: X-ray photoelectron spectroscopy (XPS) spectra of the $\mathbf{Pn_{XCB}}$ materials.

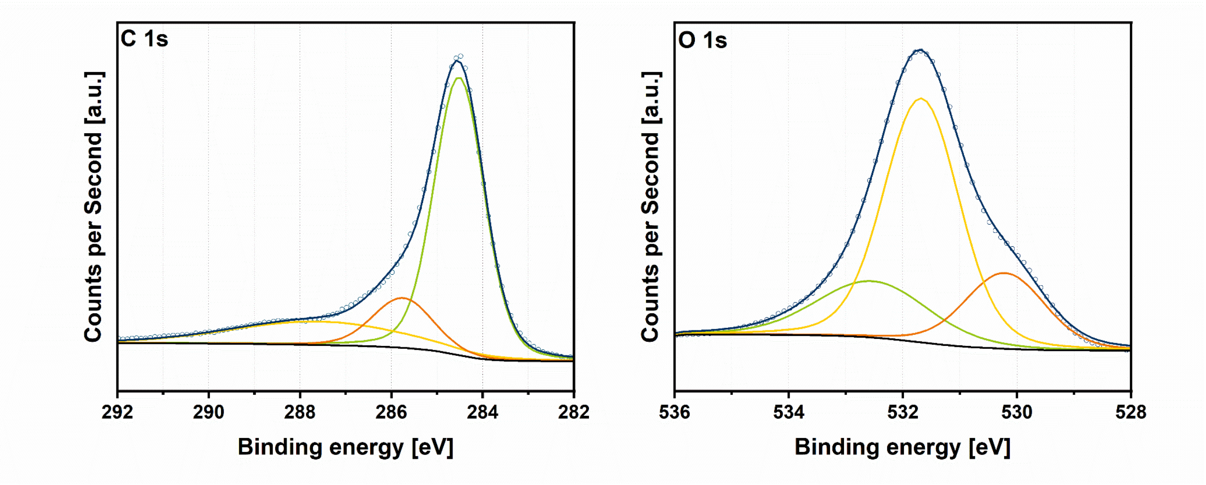

Fig. S7: C 1s and O 1s X-ray photoelectron spectroscopy (XPS) spectra of the **Pn$_{25\%CB}$** material.

Transmission electron microscopy (TEM) images coupled with energy dispersive X-ray emission spectroscopy (EDX) mappings of **Pn**

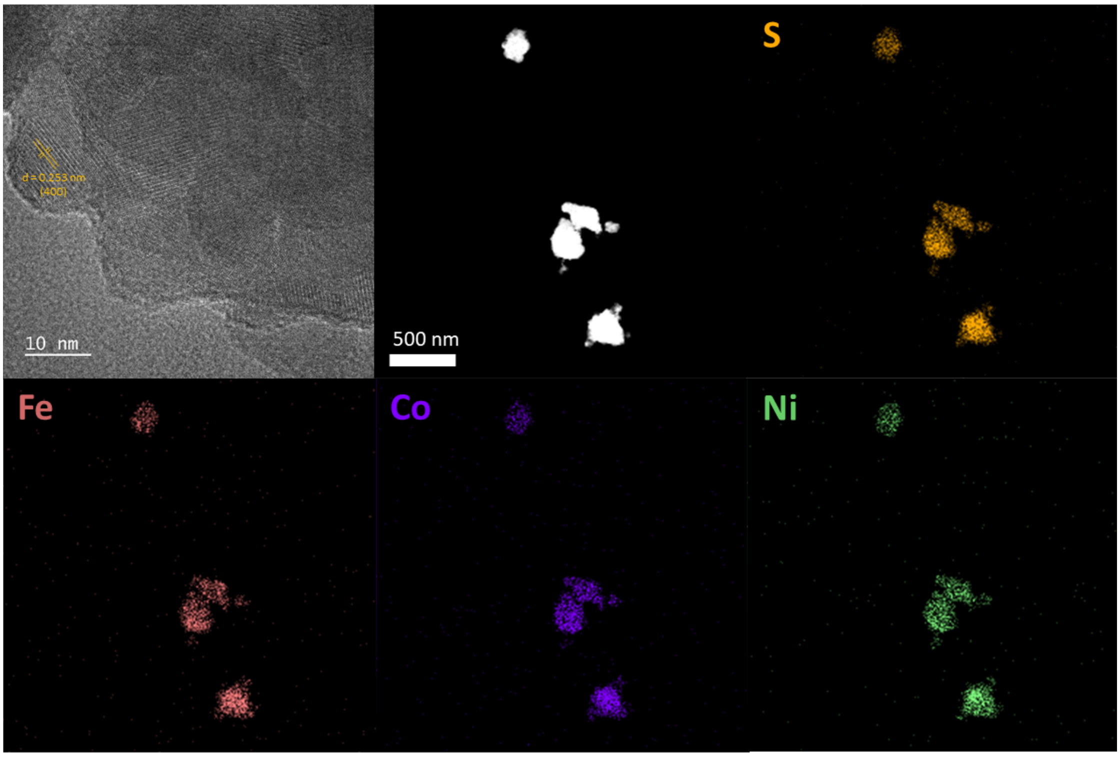

Fig. S8: High resolution TEM image of **Pn** (top left). Dark field scanning tunnelling electron microscopy (DF-STEM) image of **Pn** (top middle) including energy dispersive X-ray spectroscopy (EDX) element maps displaying a homogeneous distribution of the elements.

Transmission electron microscopy (TEM) images coupled with energy dispersive X-ray emission spectroscopy (EDX) mappings of $Pn_{10\%CB}$

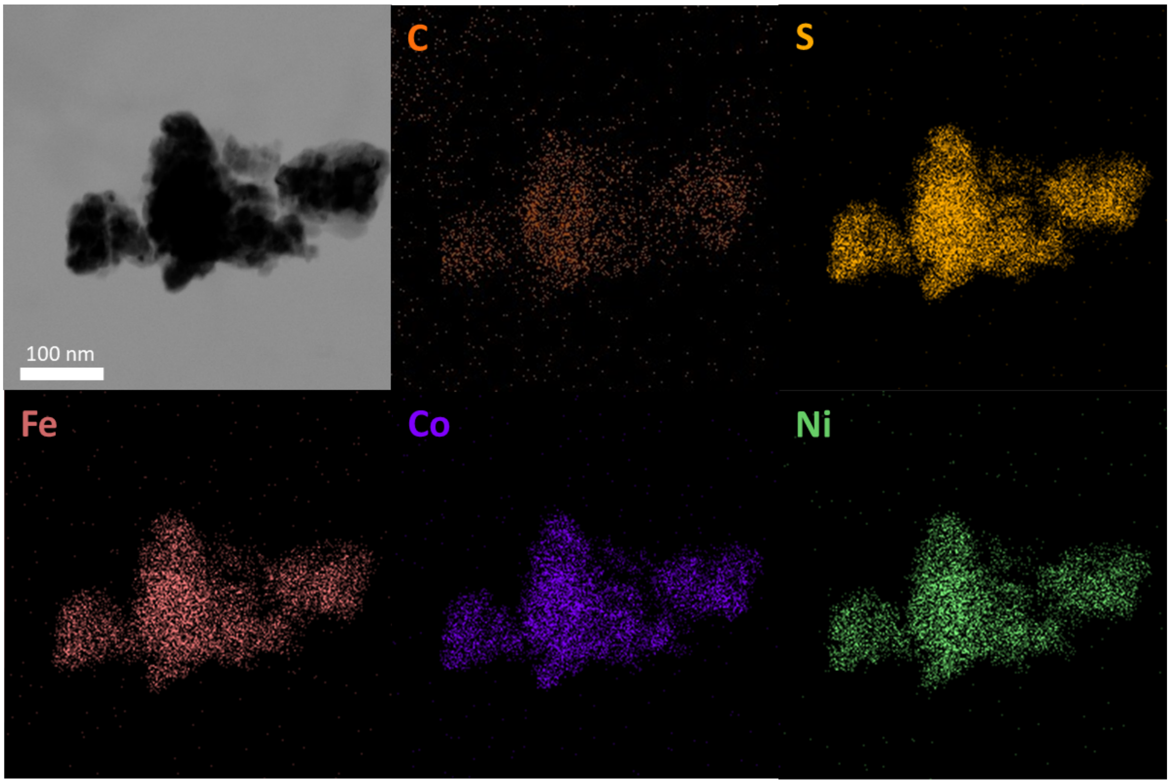

Fig. S9: Bright field scanning tunnelling electron microscopy (BF-STEM) image of **$Pn_{10\%CB}$** including energy dispersive X-ray spectroscopy (EDX) element maps displaying a homogeneous distribution of the elements

Transmission electron microscopy (TEM) images coupled with energy dispersive X-ray emission spectroscopy (EDX) mappings of $\mathbf{Pn_{50\%CB}}$

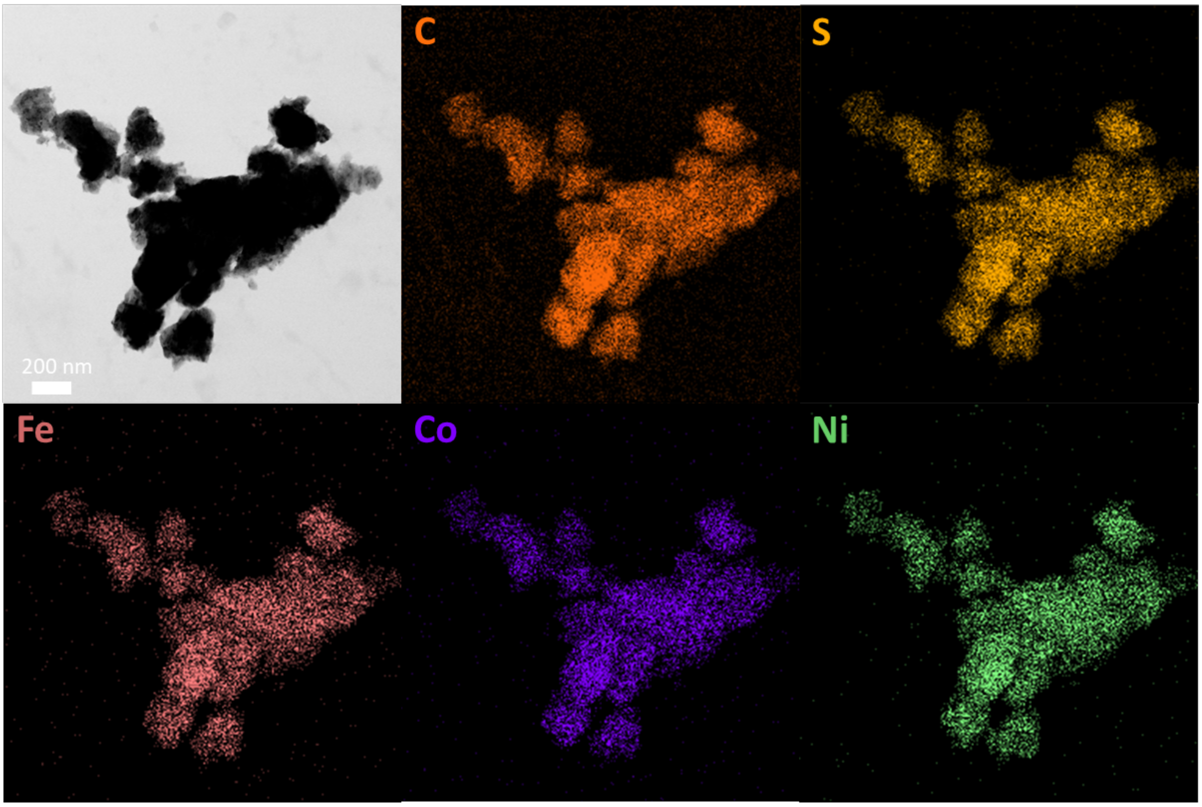

Fig. S10: Bright field scanning tunnelling electron microscopy (BF-STEM) image of $\mathbf{Pn_{50\%CB}}$ including energy dispersive X-ray spectroscopy (EDX) element maps displaying a homogeneous distribution of the elements

Laser diffraction measurements of the synthesized $\mathbf{Pn_{XCB}}$ materials

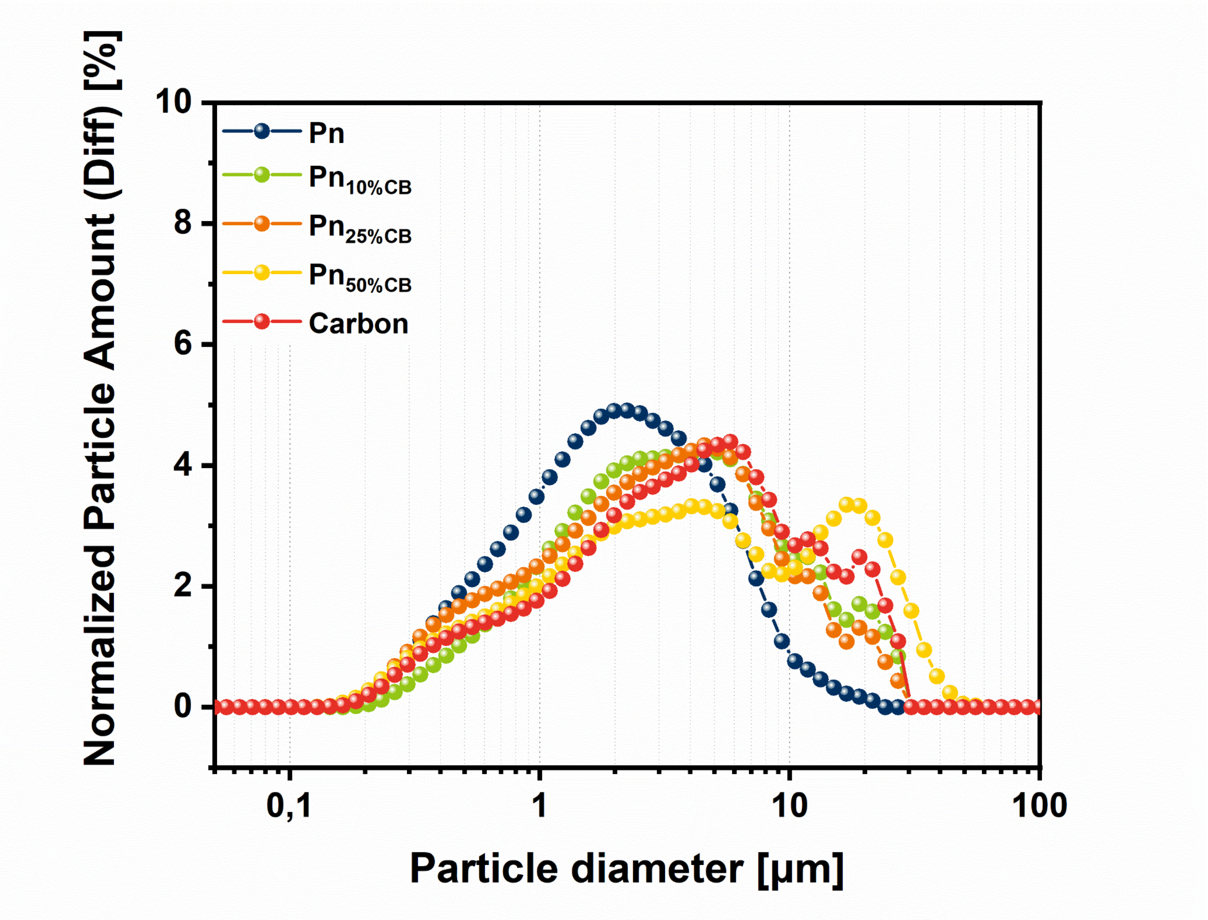

Fig. S11: Laser diffraction analysis of the synthesized $\mathbf{Pn_{XCB}}$ materials depending on the carbon amount.

Process Flexibility

One opportunity to tune the properties of the heterostructured **Pn/C** material lies in the possibility to synthesize materials with distinct **Pn** stoichiometries including mono-, bimetallic and sulfoselenide pentlandite materials. The second opportunity to tune the properties of heterostructured **Pn/C** material involves changing the carbon material. We have substituted carbon black in the trimetallic reaction mixture by graphite, carbon nanotubes (CNT) or reduced graphene oxide (rGO) for the formation of **Pn/C** materials with different carbon materials. Here, keeping the milling parameters identical to the one for the CB containing reaction mixture, the synthesis of **Pn/graphite**, **Pn/CNT** and **Pn/rGO** resulted in unfinished products. Instead, samples required an increased milling time to achieve a full **Pn**- conversion, which can be attributed to the tribilogical properties of the aforementioned carbon materials. Thus, the herein reported synthesis method enables the synthesis of pentlandite materials with a broad stoichiometry and choice of carbon materials. It is expected that other bi-, trimetallic and sulfoselenide pentlandites including other carbon sources can be prepared as well.

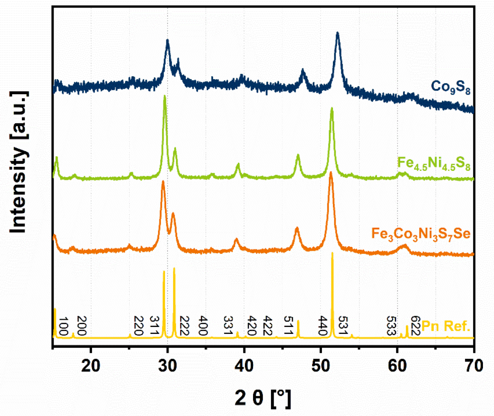

Fig. S12: Powder X-ray analysis of other Pentlandite materials with 25% CB**.**

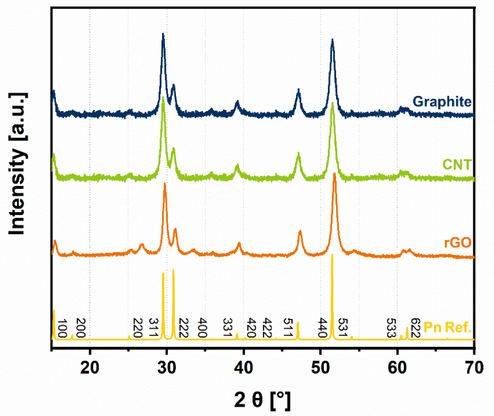

Fig. S13: Powder X-ray analysis of the synthesized $\mathbf{Pn_{25\%C}}$ materials synthesized with different carbon sources.

Environmental Analysis

To analyze the environmental impact of the presented process two different parameters were employed. While the process mass intensity (PMI, (1)) allows for comparison of the effective utilization of reactants the global warming potential (GWP, (2)) provides a metric for energetic contributions.

$$PMI = \frac{m_{process}}{m_{product}} \qquad (1)$$

$$GWP = E_{consumed} \cdot CIPK \qquad (2)$$

With $m_{process}$ being the total mass added during a process or process step, $m_{Product}$ the mass of product yielded by the process, $E_{consumed}$ the consumed energy in kWh and *CIPK* the carbon emission intensity per kilowatt-hour in $g_{CO2\text{-}equivalents}$/kWh.[1,2] The CIPK can be found for most countries and depends on how the energy is generated. For the present calculations a CIPK of 471 $g_{CO2\text{-}equivalents}$/kWh was assumed.[3] For both PMI and GWP a low number is the desired outcome. A process with PMI below 15 is generally considered as green, but should rather be considered in comparison to alternative processes.[4] The same principle applies for the GWP, where acceptable amounts strongly vary, depending on the perceived value of product.

We identified six common routes to synthesize a range of **Pn**- and heterostructured **Pn**-materials that we compared with the developed process. The most commonly used is the ampoule synthesis, followed by various coprecipitation methods, a route *via* decomposition of a dicarbamate precursor and a direct carbonization pathway.[5–9] Both, the ball mill route as well as the ampoule route have a very low process mass intensity of one, because all reactants are completely converted into product. Mixed metal coprecipitation depend highly on the starting salts. Nitrates result in the highest PMI of this group while sulfates have the lowest. Dicarbamates as precursor result in comparable PMI as ammonium sulfates at about 90. Of all compared processes, the most complex and thus environmentally malign is the direct carbonization technique. Comparing the global warming potential of the reactions caused by energy consumption ball milling is lowest as well (Fig. S14 & Table S2). The low powered electric motor of a ball mill uses only 200 Wh while the next lowest consumption is during the carbamate process that consists of a short length heating step. All other processes depend on high power furnaces with significant higher global warming potential as a result. Again, the direct carbonization has the highest GWP with 8850 $g_{CO2}/g_{Product}$. However, this process results in nanostructured carbon wires with homogeneous **Pn**-distribution across the surface. So overall, the proposed ball milling process has the lowest environmental impact if GWP and PMI are compared while being able to produce pure pentlandite as well as heterostructured samples.

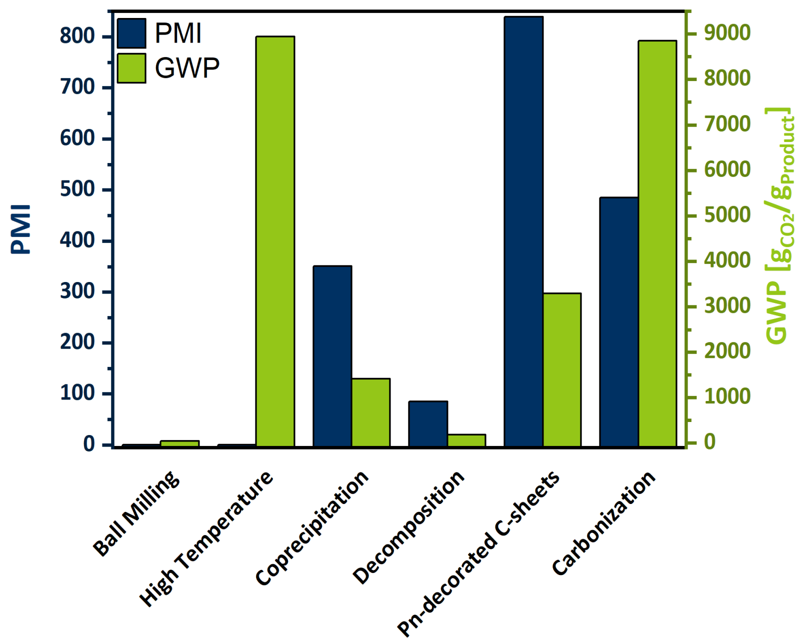

Fig. S14: Comparison of different **Pn** synthesis pathways in process mass intensity (PMI) and global warming potential (GWP). Ball milling describes the present method. High temperature abbreviates the most common ampoule syntheses. Coprecipitation was calculated from a process described by Danot et al. Decomposition of dithiocarbamates was developed by Hogarth et al. Kim et al. developed the Pn-decorated carbon sheets. Carbonization represents the decomposition route of Prussian blue analogues.

Table S2 Environmental analysis of different synthesis routes towards pentlandite materials and heterostructured pentlandites.

Process	PMI	GWP Energy (g_{CO2}/g_{CO2})
Ball Mill	1	47,0
Ampule	1	8949
Mixed metal coprecipitation from nitrates	352	1413
Mixed metal coprecipitation from ammoniumsulfates	96	1413
Mixed metal coprecipitation from sulfates	23	3062
Decomposition of dicarbamate	86	188
Carbonization of prussian blue analogues	485	8855

Cost analysis

Table S3 Resource cost analysis of pentlandite compared to platinum (all data obtained from stock prices on 13th October 2022).

Resource	Price per ton (€)	Amount needed for one ton catalyst (t)	Total price (€)
Iron	112	0,216	24
Cobalt	53114	0,228	12087
Nickel	22315	0,227	5058
Sulfur	1566	0,330	517
Pentlandite	-	-	17686
Platinum	29576757	1	29576757

Cross sectional SEM image of a catalyst coated membrane using **Pn$_{25\%CB}$** as cathodic catalyst

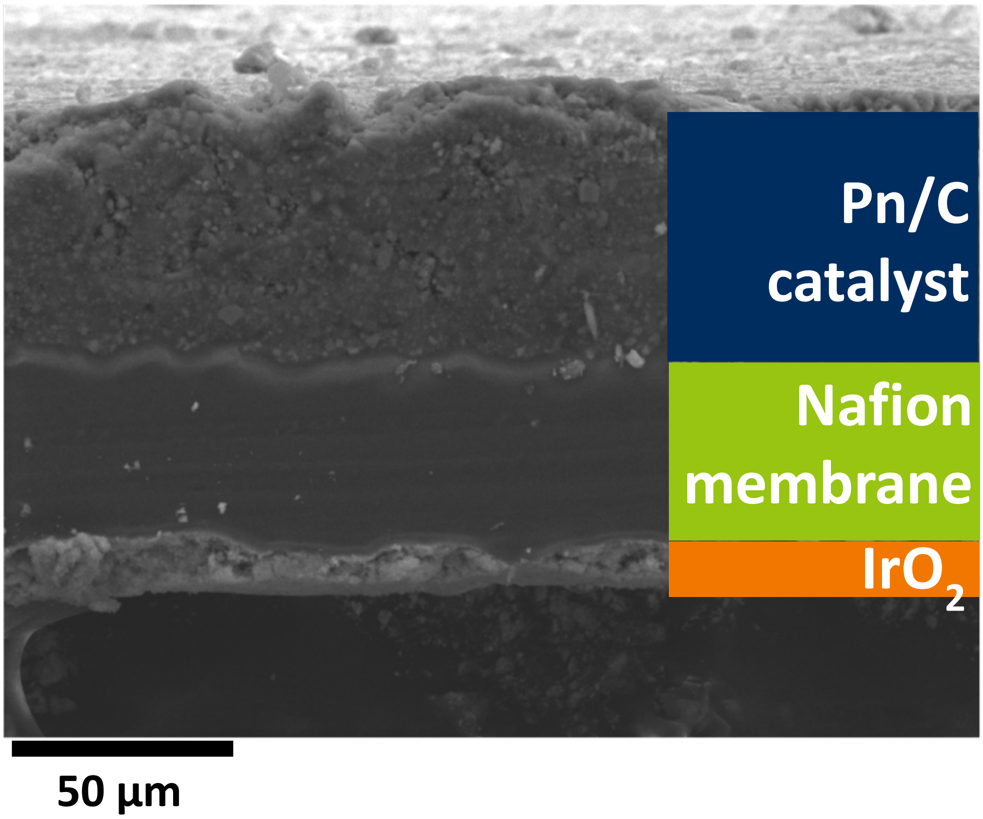

Fig. S15: Cross sectional SEM image of a catalyst coated membrane using **Pn$_{25\%CB}$** as cathodic catalyst and IrO_2 as anodic catalyst. The employed membrane is a Nafion 212.

Cross sectional SEM image of a catalyst coated membrane using $\mathbf{Pn}_{25\%graphite}$ as cathodic catalyst

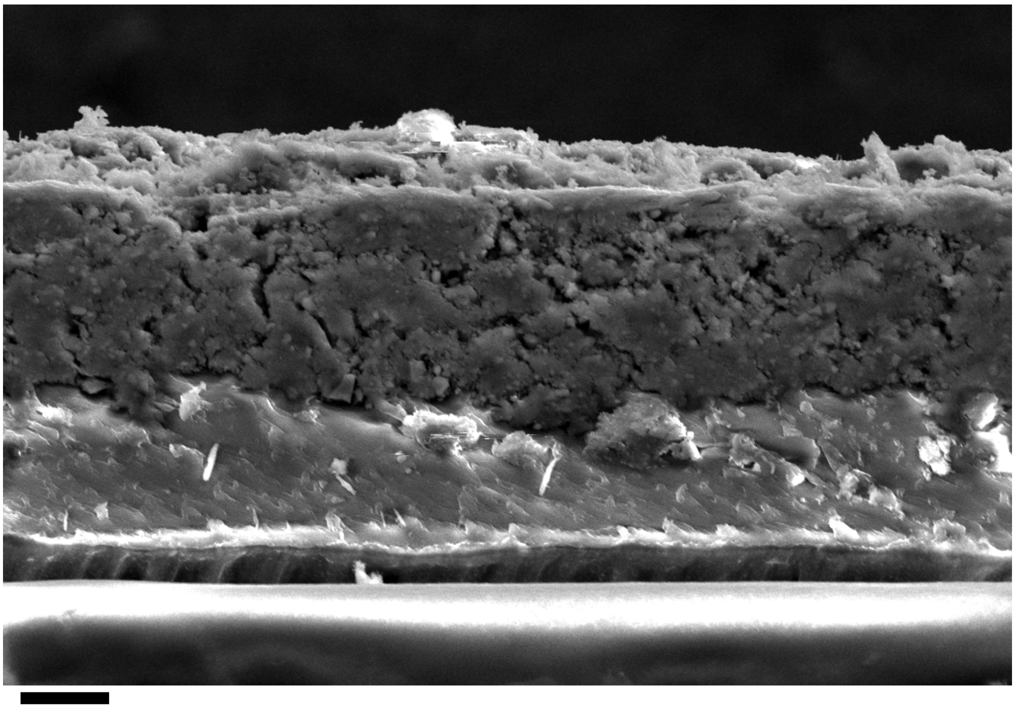

Fig. S16: Cross sectional SEM image of a catalyst coated membrane using $\mathbf{Pn}_{25\%graphite}$ as cathodic catalyst. The employed membrane is a Nafion 212. The scale is 20 µm.

Cross sectional SEM image of a catalyst coated membrane using **$Pn_{25\%CNT}$** as cathodic catalyst

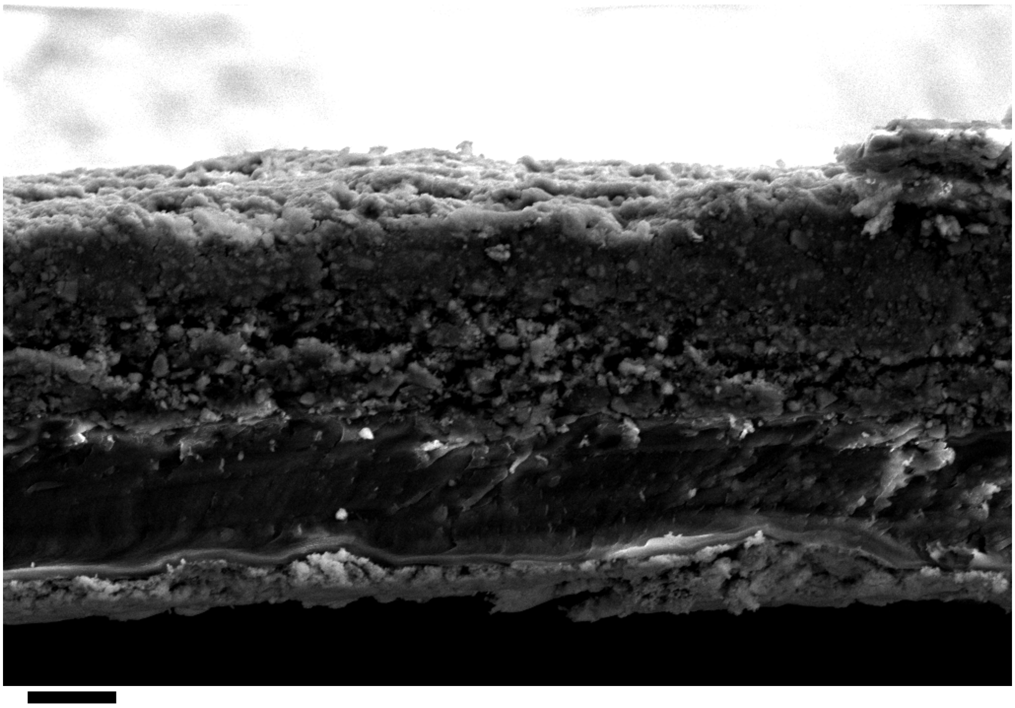

Fig. S17: Cross sectional SEM image of a catalyst coated membrane using **$Pn_{25\%CNT}$** as cathodic catalyst and IrO_2 as anodic catalyst. The employed membrane is a Nafion 212. The scale is 20 µm.

Cross sectional SEM image of a catalyst coated membrane using **Pn$_{25\%rGO}$** as cathodic catalyst

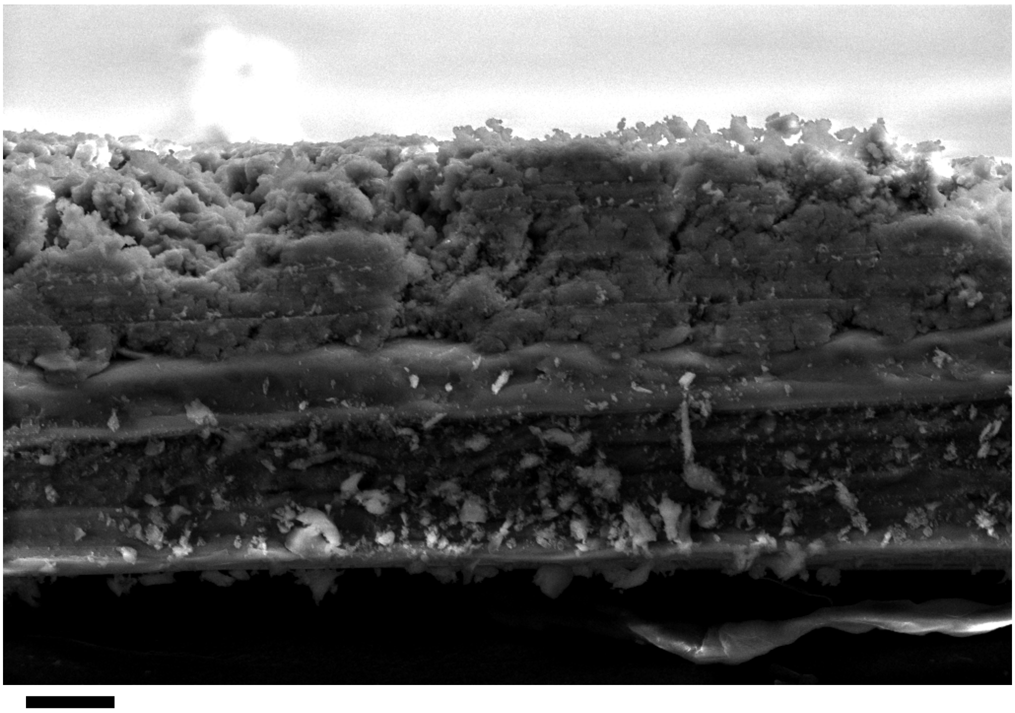

Fig. S18: Cross sectional SEM image of a catalyst coated membrane using **Pn$_{25\%rGO}$** as cathodic catalyst. The employed membrane is a Nafion 212. The scale is 20 µm.

Table of catalyst layer thicknesses of the prepared cathodes determined via SEM

Table S4: Calculated thicknesses of the cathodic catalysts layers from SEM.

	**$Pn_{25\%CB}$**	**$Pn_{25\%graphite}$**	**$Pn_{25\%rGO}$**	**$Pn_{25\%CNT}$**
Thickness [µm]	56.3 ± 4.6	50.4 ± 3.9	49.8 ± 1.5	52.3 ± 1.9

X-ray photoelectron spectroscopy (XPS) spectra of the $Pn_{25\%CB}$ CCM catalyst layer.

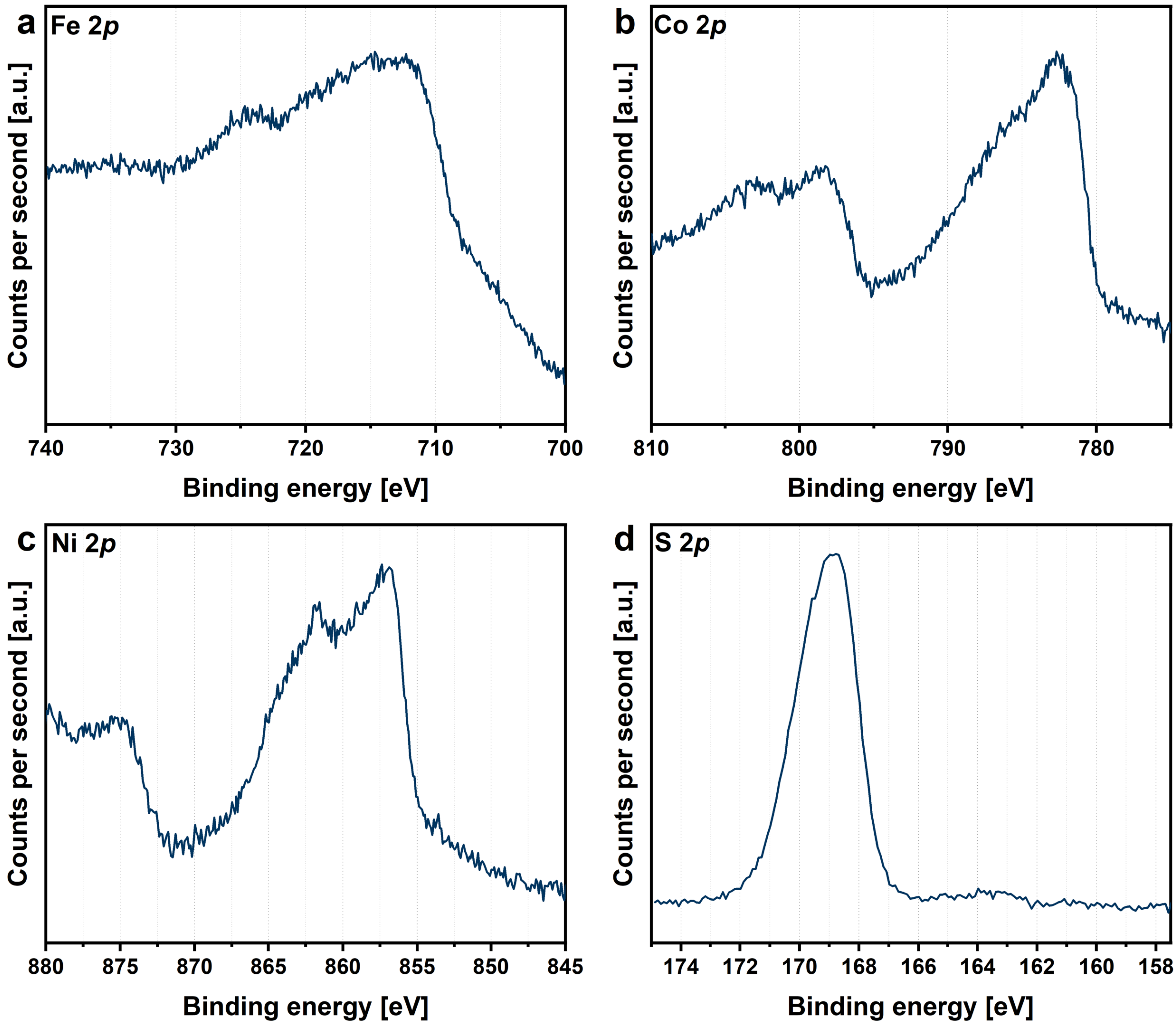

Fig. S19: X-ray photoelectron spectroscopy (XPS) spectra of the $Pn_{25\%CB}$ CCM catalyst layer.

Water vapor sorption analysis of **Pn$_{25\%CB}$**

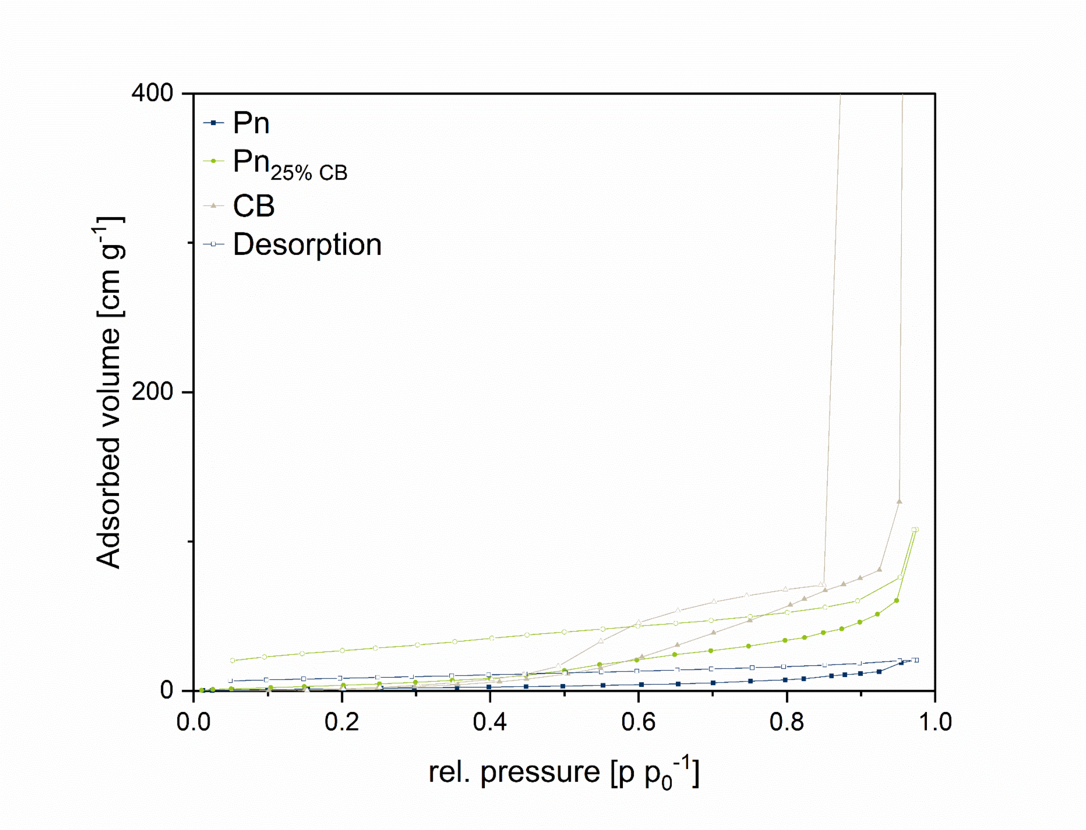

Fig. S20: Water vaporsorption isotherms of the **Pn$_{25\%CB}$** catalyst.

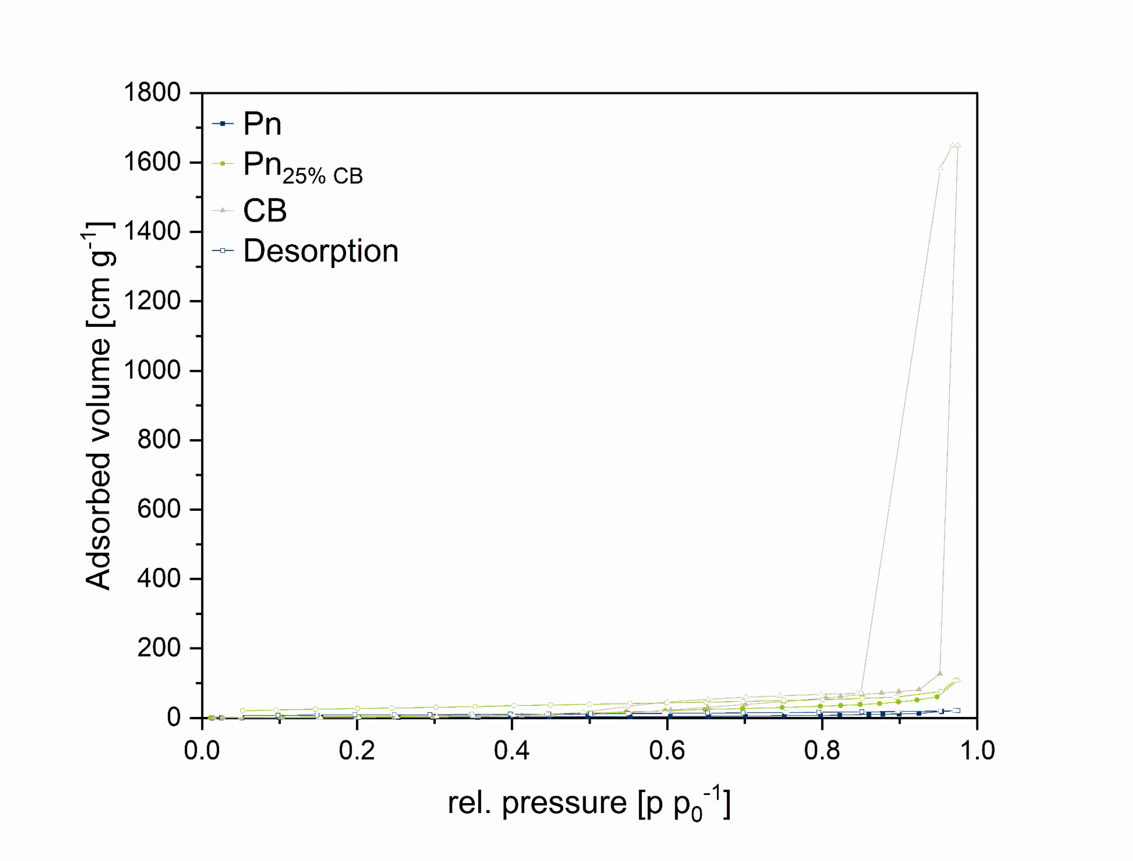

Fig. S21: Water vaporsorption isotherms of the **Pn$_{25\%CB}$** catalyst.

Water vapor sorption analysis of **$Pn_{25\%graphite}$**

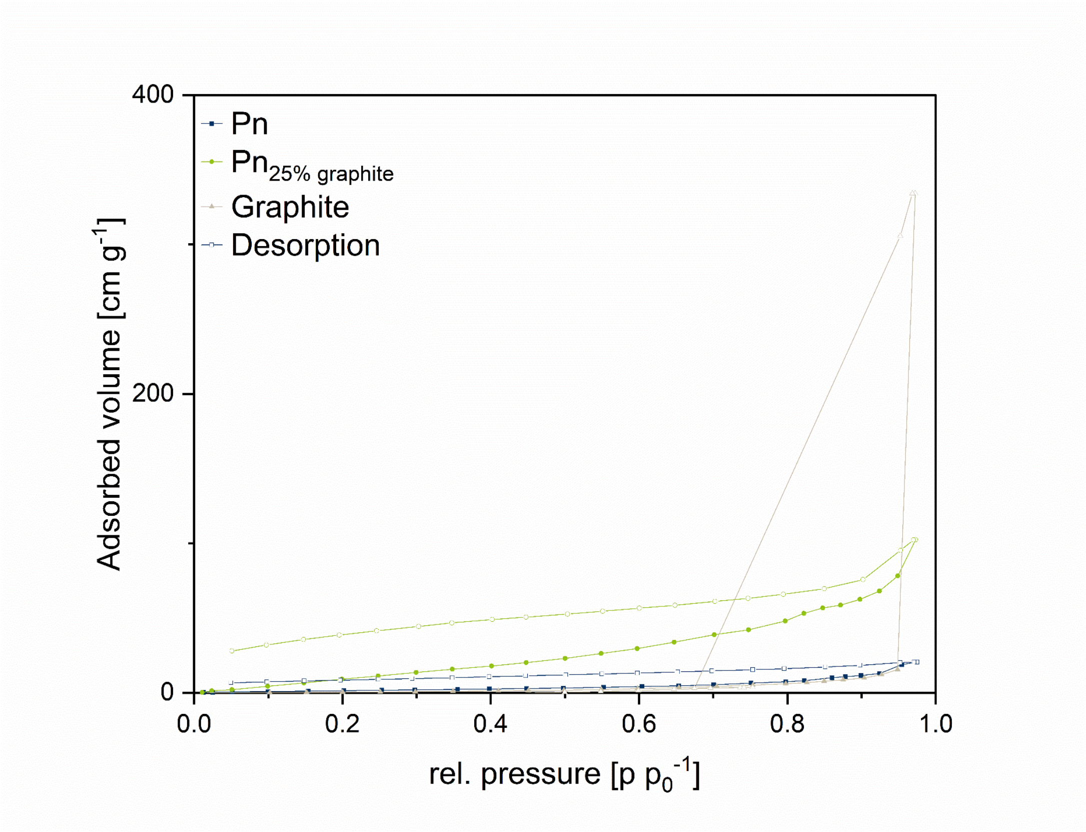

Fig. S22: Water vaporsorption isotherms of the **$Pn_{25\%graphite}$** catalyst.

Water vapor sorption analysis of **Pn$_{25\%CNT}$**

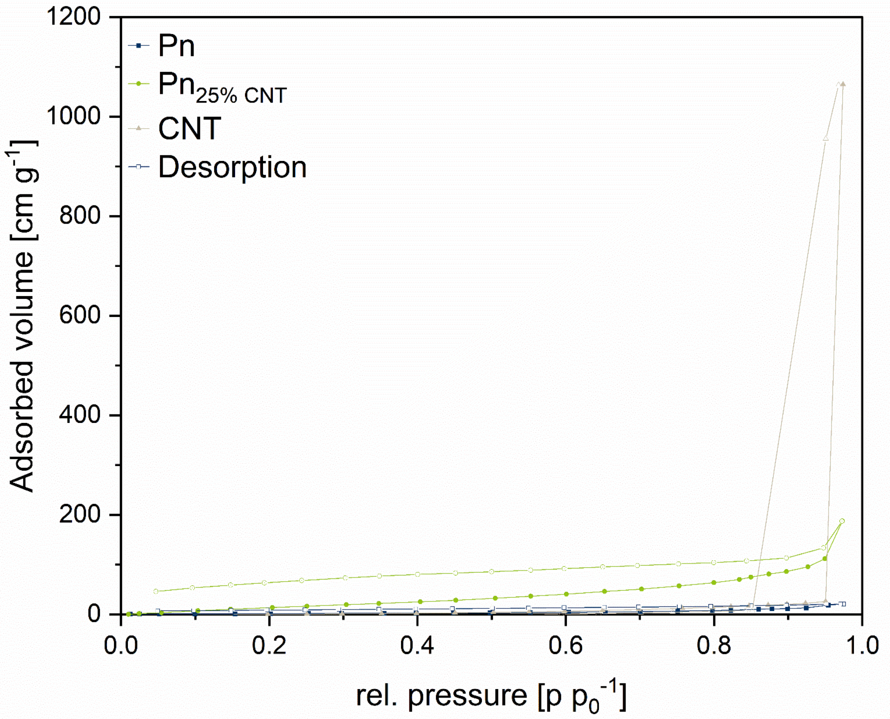

Fig. S23: Water vaporsorption isotherms of the **Pn$_{25\%CNT}$** catalyst.

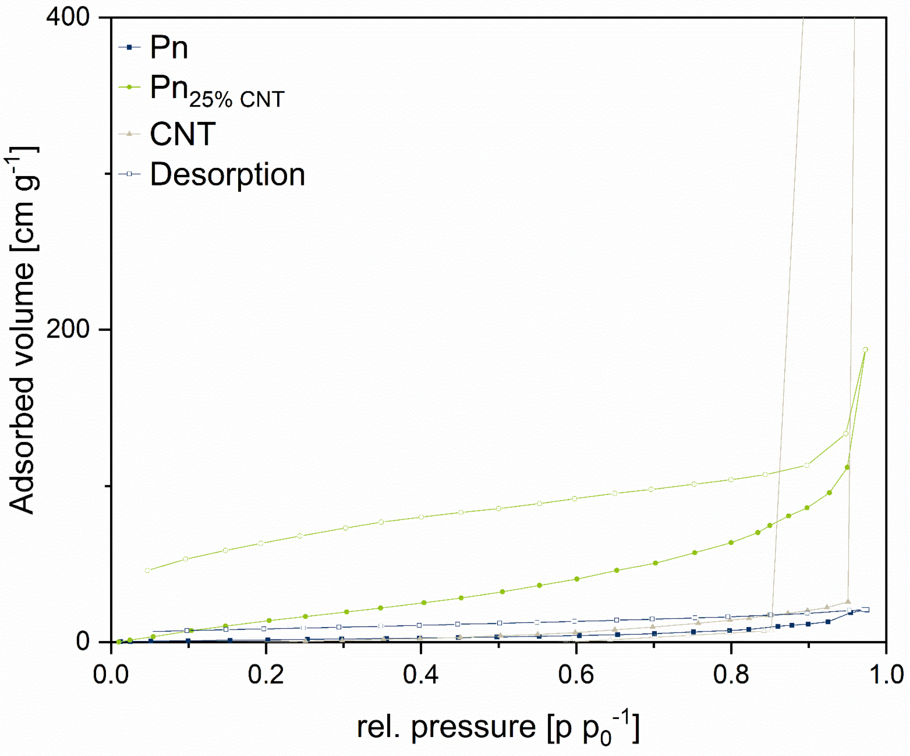

Fig. S24: Water vaporsorption isotherms of the **Pn$_{25\%CNT}$** catalyst**.**

Water vapor sorption analysis of **Pn25%rGO**

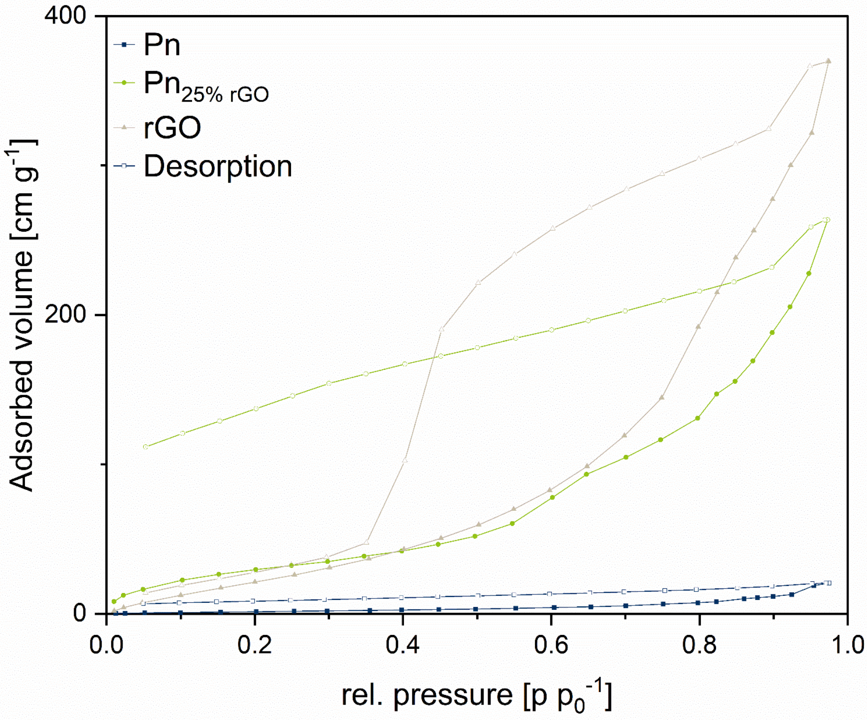

Fig. S25: Water vaporsorption isotherms of the **Pn25%rGO** catalyst.

Electrochemical MEA data

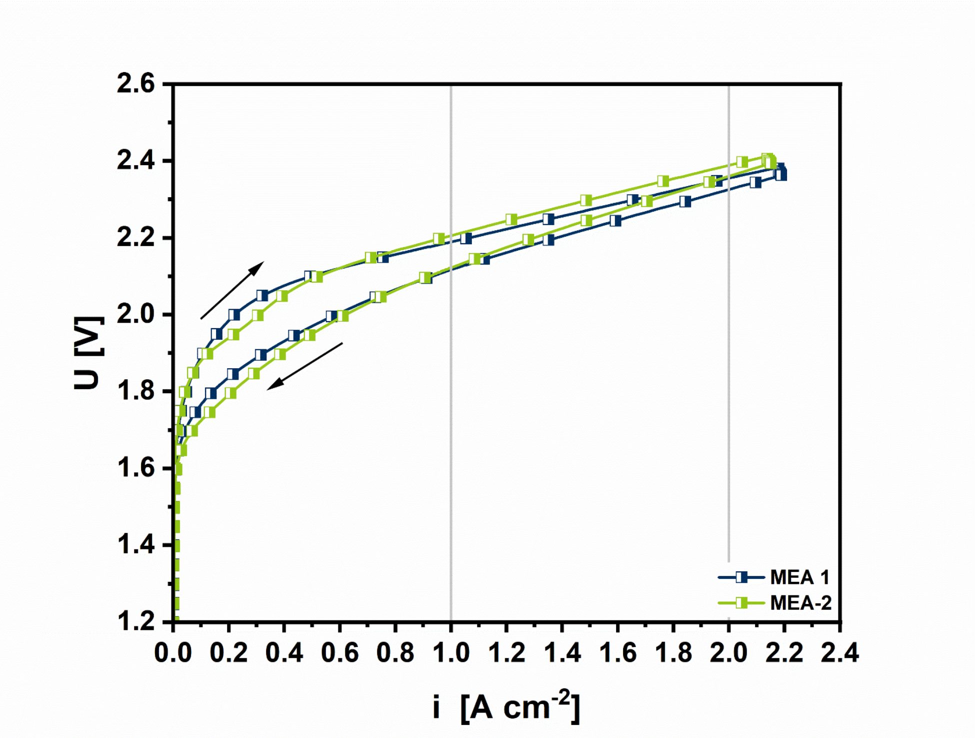

Fig. S26: CV curves of two independently prepared **$Pn_{25\%CB}$** MEAs in an in-house built PEM electrolyzer recorded with a scan rate of 0.5 mV s^{-1} at 80 °C.

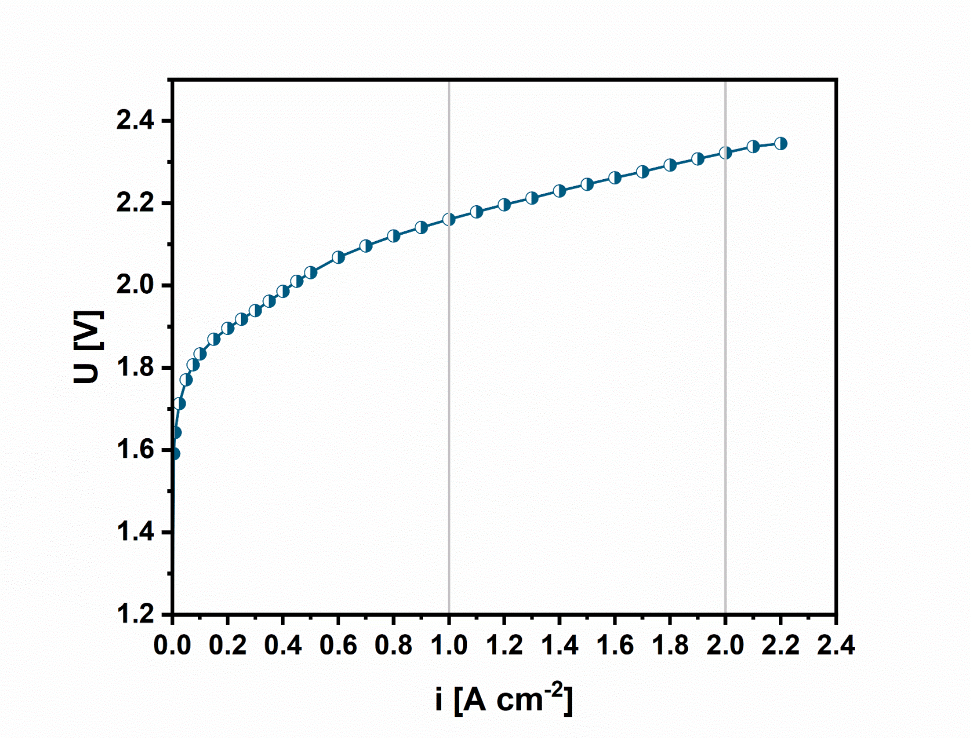

Fig. S27: Polarization curve of an independently prepared **$Pn_{25\%CB}$** MEA in an in-house built PEM electrolyzer recorded at 80 °C.

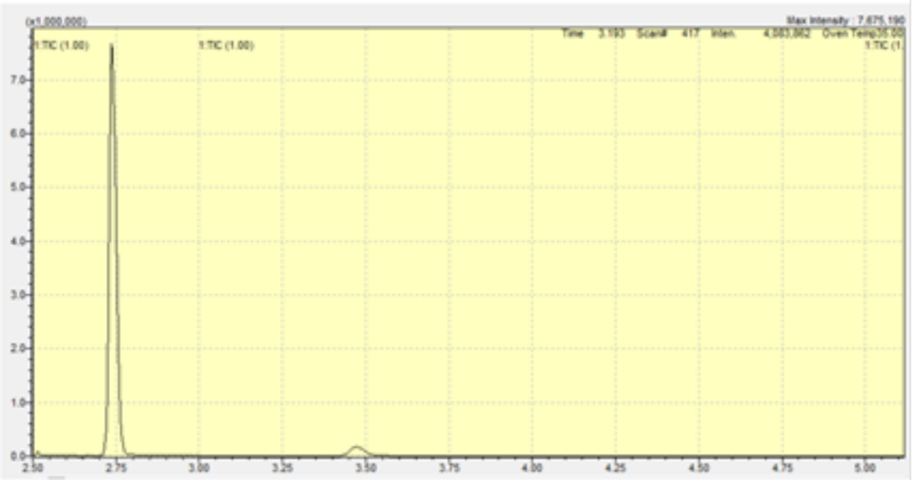

Fig. S28: Representative GC-MS-trace of product gas stream obtained with MEAs featuring the Pn-type cathodes. The Signals correspond to H_2 and O_2, respectively.

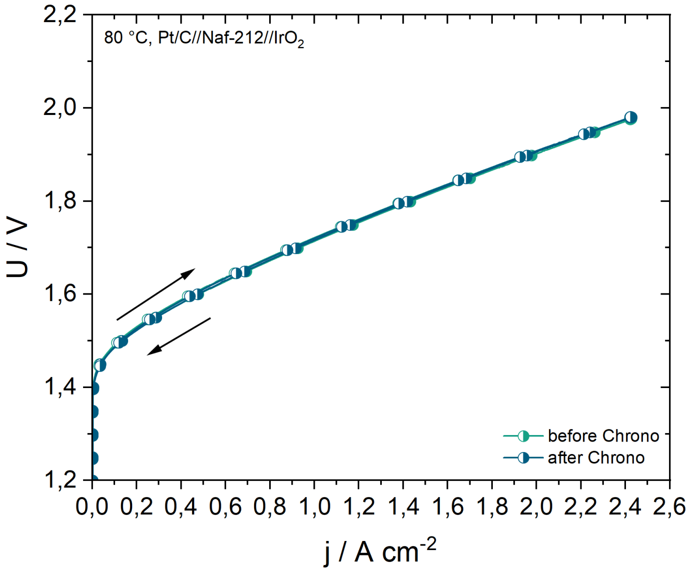

Fig. S29: Polarization curve of a benchmark Pt/C|IrO$_2$-MEA in the-house built PEM electrolyzer recorded at 80 °C.

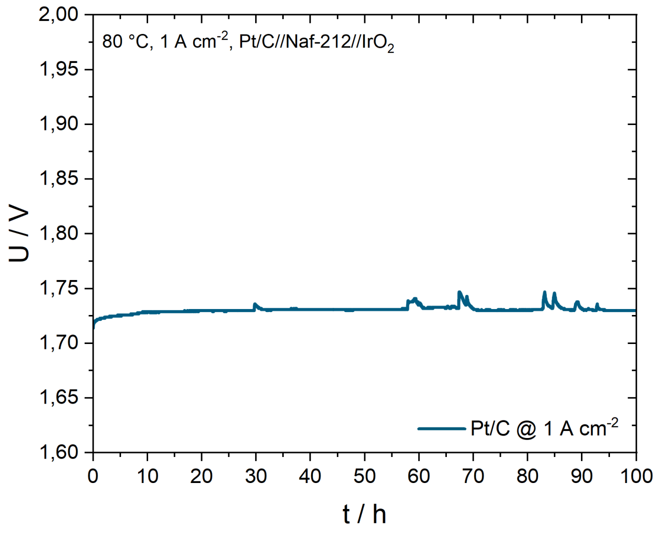

Fig. S30: Chronopotentiometry of a benchmark Pt/C|IrO_2-MEA at 80 °C for 100 h at an applied current density of 1 A cm^{-2}.

Materials

Table S5: List of materials used in this work including the distributors.

Material	Distributor
Fe	Abcr
Co	Sigma Aldrich
Ni	Abcr
S	Alfa Aesar
Se	Alfa Aesar
Carbon Black XC 72R	Cabot
Graphite	Sigma Aldrich
Carbon Nanotubes	Sigma Aldrich
rGO	Sigma Aldrich
Nafion solution (5% aliph. alcohols)	Sigma Aldrich
IrO_2	Alfa Aesar

Milling durations for obtaining distinct **Pn/C** materials

Table S6: List with milling duration for obtaining pentlandite materials with distinct carbon amounts and carbon sources.

Material	Milling duration [min]
**$Pn_{10\%C}$**	45
**$Pn_{25\%C}$**	45
**$Pn_{50\%C}$**	360
**$Pn_{25\%Graphite}$**	360
**$Pn_{25\%CNT}$**	360
**$Pn_{25\%rGO}$**	360

References

1 R. A. van Santen, ed., *Catalysis. An integrated approach*, Elsevier, Amsterdam, 2nd edn., 2000, vol. 123.

2 I. M. K. Ismail, *Langmuir*, 1992, **8**, 360–365.

3 M. Bevilacqua, C. Bianchini, A. Marchionni, J. Filippi, A. Lavacchi, H. Miller, W. Oberhauser, F. Vizza, G. Granozzi, L. Artiglia, S. P. Annen, F. Krumeich, and H. Grützmacher, *Energy Environ. Sci.*, 2012, **5**, 8608.

4 C. Jimenez-Gonzalez, C. S. Ponder, Q. B. Broxterman, and J. B. Manley, *Org. Process Res. Dev.*, 2011, **15**, 912–917.

5 A. Moro, and L. Lonza, *Transportation research. Part D, Transport and environment*, 2018, **64**, 5–14.

6 P. Icha, T. Lauf and G. Kuhs, *Entwicklung der spezifischen Kohlendioxid-Emissionen des deutschen Strommix in den Jahren 1990 - 2020. Climate Change 45/2021*, 2021.

7 E. R. Monteith, P. Mampuys, L. Summerton, J. H. Clark, B. U. W. Maes, and C. R. McElroy, *Green Chem.*, 2020, **22**, 123–135.

8 M. Al-Mamun, Y. Wang, P. Liu, Y. L. Zhong, H. Yin, X. Su, H. Zhang, H. Yang, D. Wang, Z. Tang, and H. Zhao, *J. Mater. Chem. A*, 2016, **4**, 18314–18321.

9 I. Bezverkhyy, P. Afanasiev, and M. Danot, *J. Phys. Chem. B*, 2004, **108**, 7709–7715.

10 B. Konkena, K. Junge Puring, I. Sinev, S. Piontek, O. Khavryuchenko, J. P. Dürholt, R. Schmid, H. Tüysüz, M. Muhler, W. Schuhmann, and U.-P. Apfel, *Nat. Commun.*, 2016, **7**, 12269.

11 A. Roffey, N. Hollingsworth, and G. Hogarth, *Nanoscale Adv.*, 2019, **1**, 3056–3066.

12 F. Wang, K. Li, J. Li, L. M. Wolf, K. Liu, and H. Zhang, *Nanoscale*, 2019, **11**, 16658–16666.
